# Gratitude and sleep disturbance in primary care patients: the mediating roles of health self-efficacy, health behaviors, and psychological distress

**DOI:** 10.3389/frsle.2025.1459854

**Published:** 2025-04-17

**Authors:** Heather R. Altier, Jameson K. Hirsch, Annemarie Weber, Niko Kohls, Jörg Schelling, Loren L. Toussaint, Fuschia M. Sirois, Martin Offenbächer

**Affiliations:** ^1^Department of Psychiatry and Behavioral Sciences, Johns Hopkins University School of Medicine, Baltimore, MD, United States; ^2^Department of Psychiatry and Behavioral Sciences, Quillen College of Medicine, East Tennessee State University, Johnson City, TN, United States; ^3^Ärzte der Welt e.V. [Doctors of the World], Munich, Germany; ^4^Division of Health Promotion, Faculty for Applied Natural Sciences and Health, Coburg University of Applied Sciences and Arts, Coburg, Germany; ^5^Institute of General Practice and Family Medicine, Faculty of Medicine, Ludwig Maximilian University of Munich, Munich, Germany; ^6^Department of Psychology, Luther College, Decorah, IA, United States; ^7^Department of Psychology, Durham University, Durham, United Kingdom; ^8^Gasteiner Heilstollen, Böckstein, Austria; ^9^Institute of Ecomedicine, Paracelsus Medical University, Salzburg, Austria

**Keywords:** insomnia, sleep disturbance, health behaviors, health self-efficacy, psychological distress, gratitude, primary care

## Abstract

**Introduction:**

Sleep disturbances are prevalent among primary care patients, and psychological dysfunction, including stress, anxiety, and depression, are robust contributors to poor sleep health. Yet, the presence of potential protective characteristics, such as health self-efficacy and engaging in adaptive health behaviors, may mitigate such outcomes. Gratitude (i.e., recognition and appreciation of experiences, relationships, and surroundings), a positive psychological cognitive-emotional characteristic, may serve as a catalyst of these beneficial downstream effects, given its association with improved health functioning and sleep.

**Methods:**

In a sample of primary care patients (*N* = 869, *M* age = 53; 60.7% female) from 50 urban and 30 rural practices in Germany, health self-efficacy (i.e., belief in ability to perform necessary actions to manage health) and constructive health behaviors (i.e., actions taken to modify health positively), separately and together as parallel first-order mediators, and stress, anxiety, and depression, as parallel second-order mediators, were investigated as potential serial mediators of the association between gratitude and sleep disturbances. Participants completed self-report measures in person and online.

**Results:**

Significant serial mediation was observed across models, although effects varied. In the first model, gratitude was associated with greater health self-efficacy and, in turn, to less stress (*a*_1_*d*_21_*b*_4_ = −0.019, 95% CI [−0.039, −0.002], *SE* = 0.010), anxiety (*a*_1_*d*_31_*b*_5_ = −0.026, 95% CI [−0.045, −0.008], *SE* = 0.009), and depression (*a*_1_*d*_41_*b*_6_ = −0.020, 95% CI [−0.040, −0.003], *SE* = 0.009), and fewer consequent sleep disturbances. In the second model, health behaviors, and anxiety (*a*_1_*d*_31_*b*_5_ = −0.009, 95% CI [−0.019, −0.002], *SE* = 0.004) and depression (*a*_1_*d*_41_*b*_6_ = −0.007, 95% CI [−0.016, −0.001], *SE* = 0.004), were serial mediators, but health behaviors and stress were not. In a final combined model, serial mediation occurred on two pathways, health self-efficacy and anxiety (*a*_1_*d*_41_*b*_6_ = −0.026, 95% CI [−0.046, −0.009], *SE* = 0.009), and health self-efficacy and depression (*a*_1_*d*_51_*b*_7_ = −0.019, 95% CI [−0.037, −0.003], *SE* = 0.009), and a specific indirect effect was found for health behaviors (*a*_2_*b*_4_= −0.086, 95% CI [−0.140, −0.030], *SE* = 0.026), but not self-efficacy.

**Discussion:**

Overall, gratitude was associated with reduced sleep disturbances through positive health behavior engagement, and via the serial mediation effects of greater health self-efficacy and lower psychological distress. Clinical interventions that enhance gratitude (e.g., gratitude listing or diaries), self-efficacy (e.g., disease self-management programs), or health behavior engagement (e.g., weight management programs) may promote favorable downstream effects on psychological distress and sleep disturbances among primary care patients.

## 1 Introduction

Sleep disturbances are prevalent worldwide, with 44% of adults reporting difficulty falling asleep and 35% indicating difficulty maintaining sleep (Aernout et al., 2021). Forty percent of primary care visits address sleep-related complaints (Arroll et al., 2012). In the United States, the number of outpatient ambulatory visits with sleep disturbance listed as the chief complaint increased 29% in a decade (Ford et al., 2014). Problems initiating and maintaining sleep are the defining criteria of the most prevalent sleep disorder, insomnia, which afflicts 22% of the global population and is more common (1.6:1) in females than males (Zeng et al., 2020). Insomnia symptoms occur in approximately one-third of primary care patients and difficulty maintaining sleep is the most prevalent characteristic, present in 80% of primary care patients with insomnia (Léger et al., 2010). Both oversleeping and undersleeping are associated with musculoskeletal, endocrine, respiratory, and neurological disorders, inflammation, higher cardiometabolic risk, and mortality (Åkerstedt et al., 2017; Irwin et al., 2016; Kanagasabai and Chaput, 2017; Ohayon et al., 2013; Shorofsky et al., 2019).

Given the deleterious impact of sleep disturbance on health, detection and intervention are imperative (Chattu et al., 2018). Individual-level cognitive-emotional factors are often more amenable to clinical intervention than fixed biological contributors (Hale et al., 2020). Gratitude is one such factor, and has been linked to improvements in health functioning, including sleep (Jackowska et al., 2016; Ng and Wong, 2013). Gratitude is an affective trait or emotion that encompasses appreciation for benefits received, including thankfulness for the present moment, nature, beauty, and life circumstances (McCullough et al., 2002; Wood et al., 2008). Several studies have demonstrated that gratitude can improve sleep. A systematic review of randomized controlled trials revealed that gratitude interventions (e.g., gratitude diaries, daily gratitude lists) improved subjective sleep quality in five samples, including individuals diagnosed with anxiety and depression, patients with neuromuscular disease, community adults, and college students (Boggiss et al., 2020). In these and other studies, the influence of gratitude on sleep disturbances is rarely explored without evaluating cognitive-emotional mechanisms or psychopathology. For example, in an RCT among German community members, a gratitude intervention improved gratitude and reduced worry, anxiety, depression, and insomnia symptoms post-intervention and at 3- and 6- month follow-ups (Heckendorf et al., 2019). In another RCT, individuals endorsing anxiety and depression diagnoses reported reduced stress, anxiety, depression, and sleep difficulties after completing a 3-week gratitude diary and, at 3-week follow-up, stress scores were maintained and anxiety scores had significantly improved (Southwell and Gould, 2017). Finally, among young adults, depressive symptoms explained the effect of gratitude on longer sleep duration and higher daytime energy (Alkozei et al., 2019), and among Austrian patients with rheumatic and musculoskeletal disease, gratitude was associated with less stress, anxiety, and depressive symptoms and, in turn, to better sleep quality and less functional impairment (Hirsch et al., 2021).

**Figure 1:**
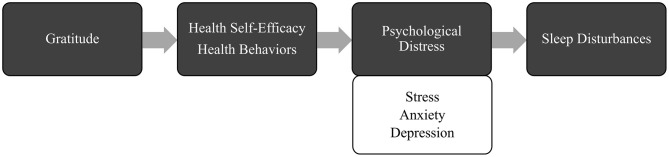
Illustration of theoretical model depicting hypothesized multiple mediation effects of gratitude on sleep disturbances via health self-efficacy, health behaviors, and psychological distress.

Extant research provides support for a beneficial effect of gratitude on amelioration of stress, anxiety, and depression, and sleep disturbances, both directly and indirectly. Yet, the mechanisms responsible for this protective influence have remained largely unexplored. In our theoretical model (see Figure 1), we propose that health self-efficacy, or belief in ability to perform health-supporting behaviors (Sirois, 2007), and engagement in wellness activities, are two potential explanatory linkages (Cousin et al., 2021). Drawing from the broaden-and-build theory (Fredrickson, 2004), gratitude may promote wellbeing by broadening one's awareness, thoughts, and behaviors over time, thereby promoting the development of resources (e.g., health, relationships, skills) that can be utilized to cope with stress (Fredrickson, 2004; Wood et al., 2010). Additionally, the coping hypothesis suggests that gratitude encourages adaptive coping strategies, such as planning and social support, which protect against stress and other adverse mental health outcomes (Wood et al., 2007). Finally, according to the positive affect hypothesis, gratitude, as a pleasant emotion, may produce additional constructive emotions that promote wellbeing and life satisfaction (Wood et al., 2010).

Consistent with these theories, prior studies independently link gratitude to health-promoting beliefs (e.g., health self-efficacy), behaviors (e.g., exercise, diet), and reduced psychological distress (DuBois et al., 2016; Grant and Gino, 2010). For example, among patients with heart failure, gratitude was associated with improved self-efficacy to maintain physical functioning and, in turn, to better medication adherence (Cousin et al., 2020), and with greater self-efficacy to preserve heart function, less depression, better sleep, and lower levels of inflammatory markers (Mills et al., 2015). Further, gratitude for one's health predicted heightened physical activity, as assessed via accelerometer, and gratitude for one's life predicted medical adherence after 6 months, among acute coronary syndrome patients (Legler et al., 2019). In primary care samples, the roles of health self-efficacy and health behavior engagement in promoting psychological and sleep health is well-established (Alfaris et al., 2015; Bluestein et al., 2010). However, no studies have integrated these pathways into a unified model, nor clarified the distinct and shared roles of cognitive-emotional and behavioral mediators in the gratitude-sleep relationship.

Given that nearly 70% of primary care visits involve psychological complaints, including depression and anxiety (Hunter and American Psychological Association, 2009), significant comorbidity exists between psychological and physical conditions (Gili et al., 2010), and emerging conditions are most often identified and treated in primary care settings (Schoen et al., 2008), it is important to identify risk and protective factors that can be targeted for clinical intervention in primary care. Gratitude is one such clinically malleable factor (Heckendorf et al., 2019; Yuan et al., 2021), with robust linkages to adaptive health outcomes.

In the current study, we examined the potential serial mediation effects of health self-efficacy and health behaviors, and stress, anxiety, and depression, on the relation between gratitude and sleep disturbances. As perceived stress, anxiety, and depression frequently co-occur and interact and, in conjunction, exacerbate sleep disturbance (Demyttenaere and Heirman, 2020; Kalmbach et al., 2018; Karing, 2021) due to shared cognitive-emotional and biological mechanisms (Boggero et al., 2017; Packard et al., 2016; Thorsteinsson et al., 2019), we examined them as parallel, rather than serial or separate, mediators in our models. We hypothesized that health self-efficacy and health behaviors, separately and together as parallel first-order mediators, and stress, anxiety, and depression, as parallel second-order mediators, would serially mediate the association between gratitude and sleep disturbances, such that greater gratitude would be associated with higher levels of health self-efficacy and/or health behaviors and, in turn, to less stress, anxiety and depressive symptoms, and sleep disturbances.

## 2 Materials and methods

Over a period of 2 months, patients from 50 urban primary care practices in the Munich area and 30 rural primary care practices in the Upper Franconia region of Germany were recruited in person by clinic staff and providers, and via informational posters, to complete a questionnaire battery. Paper surveys could be returned to the practice in person or to either study center at Coburg University of Applied Sciences or the University of Munich via postal mail, and questionnaires could also be completed online. The original study was approved by the Ethics Commission of the Faculty of Medicine at the Ludwig Maximilian University of Munich, and participation was voluntary. To ensure privacy, the Institutional Review Board waived consent requirements and no written informed consent was obtained. To participate, respondents were required to be age 18 or older, to have sufficient knowledge of German, and to possess a willingness to participate. Participants under age 18 and with severe psychiatric disorders were excluded.

### 2.1 Participants

Participants (*N* = 869) were 39.1% male (*n* = 343) and 60.7% female (*n* = 533), ranging from 20 to 92 years of age (*M* = 53.00, SD = 11.96). Race and ethnicity data were not collected, as legal restrictions and sociopolitical pressures enjoin researchers from collecting ethnoracial data in Germany and much of Western Europe (Roig, 2017; Simon, 2012). Regarding education, most participants reported having an intermediate school certificate (*n* = 287; 32.7%), followed by a high school diploma (*n* = 252; 28.7%), some high school completed (*n* = 196; 22.3%), university entrance qualification (*n* = 75; 8.5%), and other certificate (*n* = 48; 5.5%). Most participants reported one chronic medical condition (*n* = 305; 34.7%), followed by zero (*n* = 206; 23.5%), two (*n* = 182; 20.7%), three (*n* = 97; 11.0%), four (*n* = 46; 5.2%), and five or more (*n* = 41; 4.6%).

### 2.2 Measures

In addition to measures assessing this study's variables, participants responded to demographic questions (e.g., age, family composition, zip code, marital status, height/weight), meant for characterizing the sample and to serve as covariates.

#### 2.2.1 Sleep disturbances

Frequency of sleep disturbances was assessed using Item 3 from the Patient Health Questionnaire-9, which was developed to detect depressive symptoms among primary care patients (Kroenke et al., 2001). Item 3 was extracted from the German translation of the PHQ-9, developed by Gräfe et al. (2004) using internationally accepted translation methods and available on Pfizer's Patient Health Questionnaire website. Participants indicated, on a scale from 0 (not at all) to 3 (nearly every day), the frequency with which they experience “Trouble falling or staying asleep, or sleeping too much”, and a higher score indicates greater sleep difficulty. This item is considered a valid measure of sleep disturbances that can be used as a substitute for longer instruments. For example, in a study aiming to evaluate the item's utility for screening for sleep disturbances in primary care patients, the PHQ item 3 demonstrated convergent validity (*r* = 0.75, *p* < 0.001) with the Insomnia Severity Index (ISI; Morin, 1993), and a cutoff score of 1, which indicates sleep problems were present several days over the past 2 weeks, yielded the optimal balance of sensitivity (82.5%) and specificity (84.5%; MacGregor et al., 2012). Further, among German cancer patients, this item was strongly correlated with the ISI (*r* = 0.72, *p* < 0.001) and performed similarly to the ISI; the ISI and Item 3 exhibited similar correlations with the Patient Health Questionnaire-2 (PHQ-2; Kroenke et al., 2003; *r* = 0.45, 0.42), Generalized Anxiety Disorder 2-item scale (GAD-2; Kroenke et al., 2007; *r* = 0.45, 0.39), and had comparable magnitudes and patterns of association with sex, age, and tumor type (Schulte et al., 2021).

#### 2.2.2 Perceived stress

The Perceived Stress Scale–4 (PSS-4; Cohen and Williamson, 1988), German version (Stächele and Volz, 2013), was used to evaluate stress. On a Likert scale, ranging from 0 (never) to 4 (very often), respondents indicated the degree of appraised stress over the past month via statements such as, “In the last month, how often have you felt that you were unable to control the important things in your life?” and “In the last month, how often have you felt that things were going your way?” After recoding two items to indicate greater perceived stress, items are summed to generate a total score between 0 and 16. The four items comprising the scale were extracted from the original scale, the PSS-14 (Cohen et al., 1983). In the current sample, the PSS-4 demonstrated acceptable reliability (Cronbach's α = 0.73; McDonald's ω = 0.74), and acceptable (α = 0.74) to good (α = 0.84) reliability in prior health samples (Vallejo et al., 2018; Wu and Amtmann, 2013).

#### 2.2.3 Anxiety

The Generalized Anxiety Disorder 2-item scale (GAD-2; Kroenke et al., 2007) measures anxiety on a Likert scale ranging from 0 (not at all) to 3 (nearly every day), via items representing core anxiety symptoms from the Generalized Anxiety Disorder 7-item scale (GAD-7; Spitzer et al., 2006). The two items utilized were from the German translation of the GAD-7, developed by Löwe et al. (2008) using internationally accepted translation methods and downloaded from Pfizer's Patient Health Questionnaire website. Participants indicate how often over the last 2 weeks they were bothered by “Feeling nervous, anxious or on edge” and “Not being able to stop or control worrying”. The total score, which ranges from 0 to 6, is obtained by summation of item scores. Higher scores denote greater anxiety symptoms, with a cutoff of 3 providing good sensitivity and specificity to detect clinically significant anxiety symptoms (Kroenke et al., 2007), which was supported in a systematic review (*N* = 5,223) of validation studies (Plummer et al., 2016). Internal consistency reliability was good in the current sample (α = 0.80; ω = 0.82). The GAD-2 exhibited acceptable reliability and strong correlations with the GAD-7 in samples of community-dwelling adults in Germany (α = 0.72; *r* = 0.87; Hinz et al., 2017) and multiple sclerosis patients (α = 0.77; *r* = 0.94; Hughes et al., 2018).

#### 2.2.4 Depression

The Patient Health Questionnaire-2 (PHQ-2; Kroenke et al., 2003), comprised of the first two items of the original measure, the Patient Health Questionnaire-9 (Kroenke et al., 2001), was used to assess depressive symptoms over the past 2 weeks. The two items were taken from Gräfe et al.'s (2004) German translation of the PHQ-9. Respondents indicated how often they were bothered by “Little interest or pleasure in doing things” and “Feeling down, depressed, or hopeless”, on a scale ranging from 0 (not at all) to 3 (nearly every day). Responses were summed for a total score ranging from 0 to 6, with higher scores indicating greater depressive symptoms. A score of 3 is considered the cutoff for detecting clinically significant depression (Staples et al., 2019), although some research suggests a more modest cutoff of 2 (Manea et al., 2016). In the current study, reliability of the PHQ-2 was good (α = 0.79; ω = 0.83), and in a sample of German COPD inpatients, composite reliability was good (CR = 0.89; Schuler et al., 2018). Among 1,619 German primary care outpatients, the PHQ-2 was strongly correlated with the PHQ-9 (*r* = 0.87; Löwe et al., 2005).

#### 2.2.5 Gratitude

Gratitude was measured using the Gratitude Questionnaire-Six Item Form (GQ-6; McCullough et al., 2002), German translation (Personality and Assessment Group, Department of Psychology, University of Zurich, n.d.), which includes six statements (e.g., “I am grateful to a wide variety of people”; “If I had to list everything that I felt grateful for, it would be a very long list”) assessing appreciation. Responses are rated on a scale ranging from 1 (strongly disagree) to 7 (strongly agree) and are summed to compute a total score between 6 and 42, after reverse scoring two items so that higher scores denote higher gratitude levels. In the current sample, internal consistency reliability was acceptable (α = 0.77; ω = 0.77). In a meta-analysis, internal consistency reliability was acceptable in European studies (α = 0.75), and good when averaging all 74 studies (α = 0.82; Card, 2019).

#### 2.2.6 Health self-efficacy

Health self-efficacy, or the degree of confidence one possesses in their ability to engage in the necessary actions to control their health, was assessed using six items from the 8-item Health Self-Efficacy/Mastery Beliefs subscale of the Control Beliefs Inventory (HSE-CBI; Sirois, 2003a), a 26-item measure of perceived control. Two items assessing aspects of health self-efficacy incorporated in the remaining items with alternative wording were excluded to reduce participant burden. As one item is reverse scored, the omission is not expected to significantly affect internal consistency. The original measure was translated from English into German, and then back-translated to English by a panel comprising members of the research team who are bilingual native German speakers fluent in English. Inconsistencies were reconciled between the researchers. Respondents indicate on a scale ranging from 1 (strongly disagree) to 6 (strongly agree) the degree to which they agree with statements such as “I am confident that I can successfully look after my health” and “I am able to meet the challenge of following a healthy routine”. After reverse scoring two negatively worded items, the average of the six items is computed to calculate an HSE-CBI total score between 1 and 6, with a higher score indicating a greater degree of health self-efficacy. In the current study, the HSE-CBI has acceptable reliability (α = 0.77; ω = 0.73), and good reliability (α = 0.84) in two samples of community adults (Sirois, 2004). The HSE-CBI demonstrated convergent validity with the General Perceived Self-Efficacy Scale (Schwarzer and Jerusalem, 1995) among community adults (Sirois, 2003b).

#### 2.2.7 Health behaviors

Participants completed the Wellness Behaviors Inventory (WBI), a measure that assesses the frequency of engagement in health-promoting behaviors, such as exercising, relaxation, and healthy eating (Sirois, 2001). The WBI was translated into German using the same procedure as the HSE-CBI. Responses to items such as “I eat breakfast” and “I exercise for 20 continuous minutes or more, to the point of perspiration” are provided on a 5-point Likert scale ranging from 1 (less than once a week or never) to 5 (every day of the week). Although the WBI consists of 12 items, only the 10 items utilized to compute the total score were included in this study. The two excluded items refer to vitamin and supplement use and form a separate optional index of health behaviors (Sirois, 2001). Two items are reverse scored before calculating the total mean score, which ranges from 1 to 5, with higher scores denoting greater engagement in health behaviors. In the current primary care sample, the WBI had questionable internal consistency reliability (α = 0.63; ω = 0.64), consistent with prior research; in a meta-analysis of 15 undergraduate and community adult samples, reliability ranged from questionable to acceptable (α = 0.64–74; Sirois et al., 2015), likely due to its checklist status.

### 2.3 Statistical analyses

#### 2.3.1 Bivariate analyses

All analyses were conducted in R Version 2022.07.1 (R Core Team, 2022). To establish the presence of bivariate relationships before testing mediation effects, Pearson's product-moment and Spearman's rank-order correlation coefficients were calculated to assess the associations among gratitude, health self-efficacy, health behaviors, stress, anxiety, and depression, and the correlations between each of these variables and sleep disturbances were evaluated with Spearman's rank-order correlation coefficients, using the R package psych (Revelle, 2022). A multicollinearity cutoff of *r* > 0.80 between study variables was used, as associations exceeding this threshold may indicate lack of independence and would be interpreted with caution (Field, 2013).

#### 2.3.2 Serial multivariate mediation analyses

Prior to conducting the mediation analyses, skewness, kurtosis, and heteroscedasticity of the data were evaluated using base R functions, to ensure the assumptions of linear regression were met (R Core Team, 2022). All predictors were standardized (i.e., converted to z-scores) because they have no natural or common metric, allowing the relative influence of each predictor to be meaningfully compared in models containing both linear and ordinal logistic regression analyses (Agresti, 1996; Menard, 2004). Sleep disturbances, being an ordered polytomous variable, was not standardized.

Three serial mediation path analyses were conducted. In the first model, the relation between gratitude and sleep disturbances, via the potential mediating effects of health self-efficacy as a first-order mediator, and stress, anxiety, and depression, as second-order parallel mediators, was assessed. In the second model, health self-efficacy was replaced with health behaviors as the first-order mediator. Finally, in the third model, health self-efficacy and health behaviors were evaluated as parallel first-order mediators. Age, sex, education level, and number of chronic diseases were included as covariates in each model.

##### 2.3.2.1 Analytic procedure

As the outcome variable, sleep disturbances, is not continuous and, instead, was measured using one item with four levels, the final regression equation of the serial mediation model (i.e., sleep disturbances regressed on gratitude and all mediators and covariates) was calculated using cumulative odds ordinal logistic regression with the VGAM package (Yee, 2015). All other pathways of the serial mediation model were analyzed using ordinary least squares linear regression, via the stats package (R Core Team, 2022).

For the ordinary least squares regression steps of each model, a change of one standard deviation in the predictor is associated with a change of β standard deviations in the endogenous variable. Regarding the ordinal logistic regression step of each model, a one standard deviation difference in the predictor is associated with a β-unit difference in ordered logits of sleep disturbance frequency (Menard, 2004). To simplify interpretation of the ordinal logistic regression results, the standardized coefficients were converted to odds ratios (*OR*), so that for each one standard deviation increase in the predictor, the odds of being more likely to endorse a higher frequency of sleep disturbances is multiplied *OR* times, holding constant all other variables.

Regarding the final step of each model, ordinal logistic and multinomial logistic regression models were compared, using the likelihood ratio test, to determine which type of model fit the data better, via the VGAM package (Yee, 2015). Next, the proportional odds assumption of ordinal logistic regression (i.e., stating each predictor has an equivalent effect, or parallel slopes, across all levels of the outcome variable; McCullagh, 1980) was evaluated, using likelihood ratio tests in the ordinal package (Christensen, 2019). If the assumption was violated, a partial proportional odds model was fit, which relaxes the proportional odds assumption and allows multiple regression coefficients only for predictors that have differential effects on the levels of the outcome variable (Peterson and Harrell, 1990).

Subsequently, non-significant covariates were removed from the linear and ordinal logistic regression analyses in a backward stepwise manner to preserve power, unless their inclusion improved overall model fit (Faraway, 2015). Finally, a function was written to calculate all mediation effects for each model, by multiplying coefficients for each path (e.g., the serial mediation effect of gratitude on sleep through health self-efficacy and stress is calculated as *a*_1_*d*_31_*b*_5_). As each model used standardized regression coefficients and continuous mediators, the mediated effect may be obtained by multiplying coefficients (i.e., using the product of coefficients method) from both ordinary least squares and ordinal logistic regression pathways and bootstrapping the confidence intervals (MacKinnon et al., 2007). Bootstrapping using 10,000 simulated samples and 95% confidence intervals, per the percentile method, was utilized in each model, to estimate the sampling distribution of the mediation effects. All *p*-values for the regression coefficients were adjusted using the p.adjust function in the stats package (R Core Team, 2022), applying the False Discovery Rate (FDR) method (Benjamini and Hochberg, 1995) to control for multiple comparisons across models. Pathways were considered statistically significant if the FDR-adjusted *p*-values were below the conventional threshold of 0.05. To correct the confidence intervals for the mediation effects, the False Coverage Rate (FCR) method was applied at a threshold of 0.97, which was calculated using the proportion of ratio of null effects to total effects across models. This method controls for false coverage in the estimation of CIs, reducing the risk of obtaining misleading conclusions about significance (Benjamini and Yekutieli, 2005). Mediation effects were considered statistically significant if the corrected 97% CIs did not include zero. Finally, multicollinearity for all predictors in each model was assessed by calculating the variance inflation factor (VIF) with the R package car (Fox and Weisberg, 2019). The VIF of each coefficient should be < 5 (Hair et al., 2019), and ideally around 1 (Akinwande et al., 2015).

Of note, path analysis was determined to be a more appropriate statistical approach than structural equation modeling (SEM) because we were interested in the relationships between variables and the mediation pathways, rather than any latent constructs or the measurement properties of the scales, and our one-item outcome variable precludes the use of comprehensive latent SEM. Further, the robust estimators available in R (maximum likelihood, weighted least squares mean and variance), cannot be used with bootstrapping for models with ordinal outcome variables, limiting methods for confidence interval estimation, and cannot be modeled to allow multiple slopes for a predictor when the proportional odds assumption is violated (Rosseel, 2012). However, as a supplementary analysis, we conducted SEM to obtain overall fit statistics for each model, dichotomizing the outcome variable and specifying psychological distress as a latent variable comprised of stress, anxiety, and depression. More details regarding the procedure and results can be found in the Supplementary material.

## 3 Results

### 3.1 Bivariate analyses

Pearson's bivariate and Spearman's rank-order correlation analyses indicated that gratitude was positively correlated with health self-efficacy (*r* = 0.27, *p* < 0.001) and health behaviors (*r* = 0.09, *p* < 0.01), and negatively related to stress (*r* = −0.17, *p* < 0.001), anxiety (*r* = −0.12, *p* < 0.001), depression (*r* = −0.15, *p* < 0.001), and sleep disturbances (*r* = −0.10, *p* < 0.01). Similarly, health self-efficacy and health behaviors were positively related to each other (*r* = 0.30, *p* < 0.001) and negatively related to stress, anxiety, depression, and sleep disturbances (all *p* < 0.001). Bivariate correlations between all continuous and ordinal study variables are provided in Table 1. No correlations approached or exceeded the multicollinearity cutoff of *r* > 0.80.

**Table 1:** Means, standard deviations, and bivariate correlations of study variables.

	**Mean (*SD*)**	**HSE**	**HB**	**Stress**	**Anxiety**	**Depression**	**Sleep^a^**	**Age**	**Education^a^**	**Conditions**
GS	33.44(6.53)	0.27^∧^	0.09^#^	−0.17^∧^	−0.12^∧^	−0.15^∧^	−0.10^#^	−0.07^*^	0.17^∧^	−0.16^∧^
HSE	4.50(0.75)	−	0.30^∧^	−0.31^∧^	−0.20^∧^	−0.23^∧^	−0.25^∧^	−0.07	0.23^∧^	−0.28^∧^
HB	3.55(0.65)	−	−	−0.21^∧^	−0.13^∧^	−0.16^∧^	−0.23^∧^	0.18^∧^	0.06	−0.07^*^
Stress	5.81(3.09)	−	−	−	0.49^∧^	0.51^∧^	0.29^∧^	−0.04	−0.18^∧^	0.28^∧^
Anxiety	1.35(1.42)	−	−	−	−	0.62^∧^	0.31^∧^	−0.05	−0.05	0.26^∧^
Depression	1.28(1.38)	−	−	−	−	−	0.29^∧^	−0.06	−0.11^∧^	0.32^∧^
Sleep^a^	1.19(0.97)	−	−	−	−	−	−	0.06	−0.12^∧^	0.28^∧^
Age	53.00(11.96)	−	−	−	−	−	−	−	−0.21^∧^	0.25^∧^
Education^a^	−	−	−	−	−	−	−	−	−	−0.20^∧^
Conditions	1.79(1.50)	−	−	−	−	−	−	−	−	−

GS, Gratitude: Gratitude Questionnaire–6; HSE, Health self-efficacy: Health Self-Efficacy/Mastery Beliefs subscale, Control Beliefs Inventory; HB, Health behaviors: Wellness Behaviors Inventory; Stress, Perceived stress: Perceived Stress Scale–4; Anxiety, Generalized Anxiety Disorder 2-item scale; Depression, Patient Health Questionnaire–2; Sleep, Sleep disturbances: Patient Health Questionnaire–9, Item 3.

Covariates: Age = participant age; Education = participant education level; Conditions = number of chronic medical conditions.

^a^Spearman's rank-order correlation coefficients.

^*^p < 0.05.

^#^*p* < 0.01.

^∧^*p* < 0.001.

### 3.2 Serial multivariate mediation analyses

Examination of skewness, kurtosis, and heteroscedasticity indicated the assumptions of linear regression were met. Table 2 shows the frequencies of the ordinal outcome variable, sleep disturbances, by response category, and normality data for the item.

**Table 2:** Frequencies, skewness, and kurtosis for sleep disturbances.

**Response**	**Frequency**		**Skewness**		**Kurtosis**	
	***n***	***%***	**Value**	***SE***	**Value**	***SE***
Not at all	212	24.6				
Several days	396	45.9				
More than half the days	131	15.2				
Nearly every day	123	14.3				
Total	862	100	0.56	0.08	−0.60	0.17

Skewness and kurtosis values indicate data are normally distributed.

#### 3.2.1 Model 1

Regarding model 1, the ordinal logistic regression model fit the data significantly better than the multinomial model ($\chi_{\left(10\right)}^{2}$ = 538.92, *p* ≤ 0.001) and was more parsimonious (i.e., contained the fewest regression coefficients). However, the proportional odds assumption was violated for health self-efficacy ($\chi_{\left(2\right)}^{2}$ = 7.312, *p* = 0.026). Therefore, a partial proportional odds model incorporating three regression coefficients for health self-efficacy was used.

**Figure 2:**
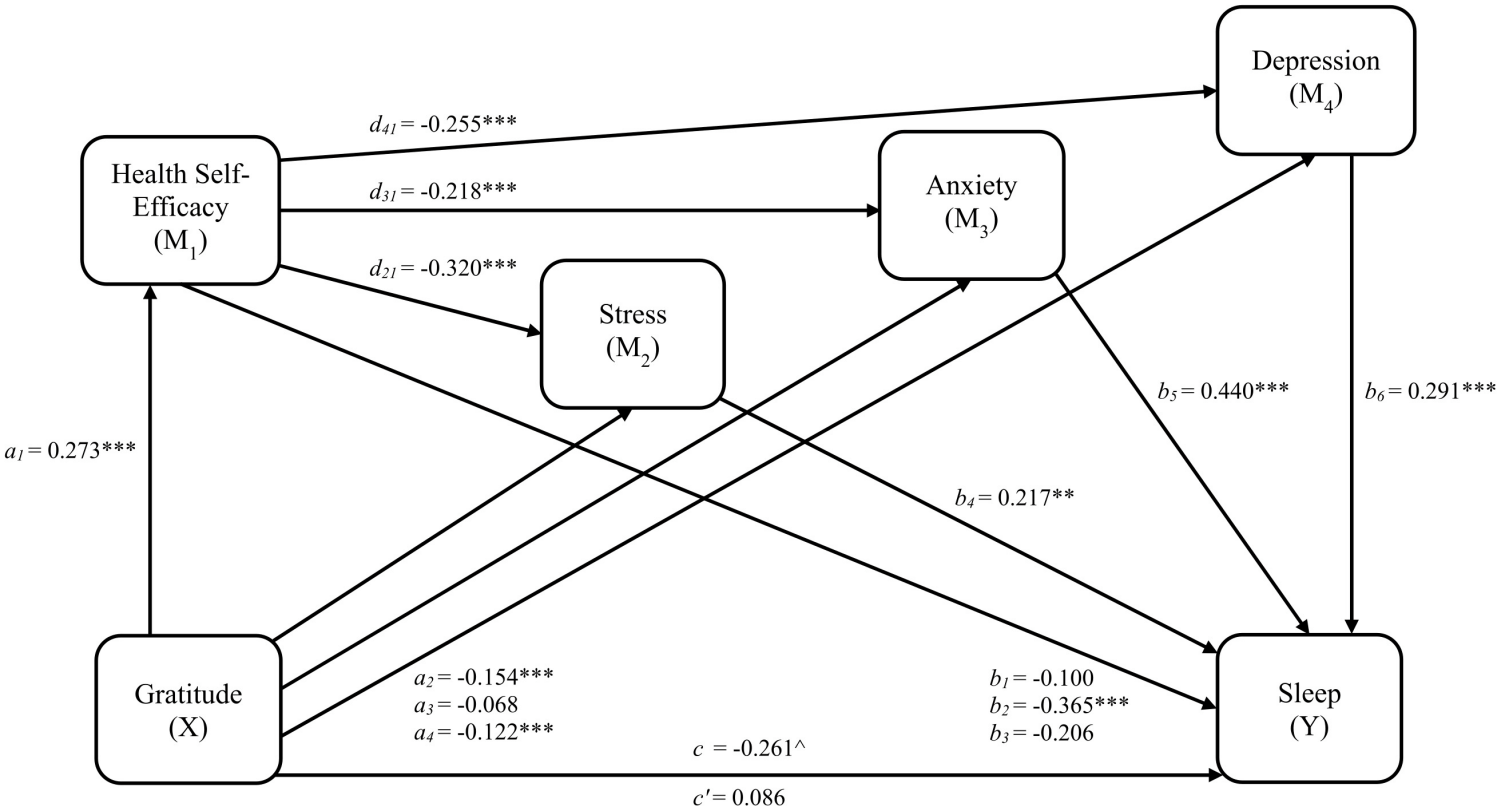
Serial indirect effects Model 1 for gratitude, health self-efficacy, psychological distress, and sleep disturbances. *a*_1_, *a*_2_, *a*_3_, *a*_4_ = regression coefficients for gratitude to health self-efficacy, stress, anxiety, and depression, respectively. *b*_1_, *b*_2_, *b*_3_ = regression coefficients for health self-efficacy to each threshold of sleep disturbances scores (0|1, 1|2, and 2|3, respectively). *b*_4_, *b*_5_, *b*_6_ = regression coefficients for stress, anxiety, and depression, respectively, to sleep disturbances. *d*_21_, *d*_31_, *d*_41_ = regression coefficients for health self-efficacy to stress, anxiety, and depression, respectively; *c* = total effect (gratitude related to sleep disturbances); *c*' = direct effect (gratitude related to sleep disturbances accounting for health self-efficacy and psychological distress). **p < 0.01; ***p < 0.001. *p*-values are FDR-adjusted. ^∧^Significance indicated by 95% CI that does not cross zero.

Linear and ordinal logistic regression results and fit statistics for each pathway, including significant covariates identified through backward stepwise selection, are provided in Table 3. Variance inflation factors are also provided in Table 3, and no predictors exceed the multicollinearity cutoff. Regression results excluding covariate effects are illustrated in Figure 2. Gratitude was related to higher health self-efficacy (β = 0.273, 95% CI [0.209, 0.337], *SE* = 0.032, *t* = 8.412, *p*_*adjusted*_ < 0.001) which was, in turn, related to lower stress (β = −0.320, 95% CI [−0.387, −0.253], *SE* = 0.034, *t* = −9.413, *p*_*adjusted*_ < 0.001), anxiety (β = −0.218, 95% CI [−0.288, −0.147], *SE* = 0.036, *t* = −6.078, *p*_*adjusted*_ < 0.001), and depression (β = −0.255, 95% CI [−0.322, −0.189], *SE* = 0.034, *t* = −7.530, *p*_*adjusted*_ < 0.001). A one standard deviation increase in stress was associated with a 24% greater likelihood of endorsing a higher frequency of sleep disturbances (*OR* = 1.242, 95% CI [1.091, 1.414], *p*_*adjusted*_ = 0.002), while equivalent increases in anxiety (*OR* = 1.553, 95% CI [1.357, 1.778], *p*_*adjusted*_ < 0.001) and depression (*OR* = 1.337, 95% CI [1.162, 1.540], *p*_*adjusted*_ < 0.001) were associated with 55% and 34% greater likelihood of sleep problem frequency, respectively.

**Table 3:** Regression coefficients of serial indirect effects Model 1 for gratitude, health self-efficacy, psychological distress, and sleep disturbances, with covariates.

**Effect^a^**	**β**	***SE***_β_	**95% CI β**		***t***	***p***	***Adj. p***	***VIF***	**Fit statistics**
			*LL*	*UL*					
HSE									*R*^2^ = 0.201;*R*^2^_adjusted_ = 0.197*F*_(4,807)_ = 50.74,*p* < 0.001
(Intercept)	− 0.361	0.179	− 0.713	− 0.010	− 2.018	0.044	**0.055**		
Gratitude	0.273	0.032	0.209	0.337	8.412	< 0.001	< 0.001	1.052	
Age	0.008	0.003	0.003	0.014	2.938	0.003	0.005	1.158	
Education	0.077	0.023	0.031	0.123	3.300	0.001	0.002	1.074	
Conditions	− 0.196	0.023	−0.242	−0.151	− 8.488	< 0.001	< 0.001	1.181	
Stress									*R*^2^ = 0.256;*R*^2^_adjusted_ = 0.252*F*_(4,814)_ = 69.96,*p* < 0.001
(Intercept)	0.449	0.139	0.176	0.722	3.226	0.001	0.002		
HSE	− 0.320	0.034	− 0.387	− 0.253	− 9.413	< 0.001	< 0.001	1.239	
Gratitude	− 0.154	0.033	− 0.218	− 0.090	− 4.713	< 0.001	< 0.001	1.141	
Age	− 0.014	0.003	− 0.019	− 0.008	− 4.927	< 0.001	< 0.001	1.149	
Conditions	0.147	0.023	0.102	0.193	6.335	< 0.001	< 0.001	1.300	
Anxiety									*R*^2^ = 0.178;*R*^2^_adjusted_ = 0.172 *F*_(5,816)_ = 35.13, *p* < 0.001
(Intercept)	0.128	0.159	− 0.184	0.439	0.805	0.421	0.421		
HSE	− 0.218	0.036	− 0.288	− 0.147	− 6.078	< 0.001	< 0.001	1.242	
Gratitude	− 0.068	0.034	− 0.136	− 0.001	− 1.987	0.047	**0.057**	1.145	
Age	− 0.011	0.003	− 0.017	− 0.006	− 3.953	< 0.001	< 0.001	1.164	
Sex (female)	0.255	0.066	0.125	0.386	3.844	< 0.001	< 0.001	1.037	
Conditions	0.182	0.024	0.134	0.230	7.455	< 0.001	< 0.001	1.301	
Depression									*R*^2^ = 0.252;*R*^2^_adjusted_ = 0.248*F*_(5,816)_ = 55.09,*p* < 0.001
(Intercept)	0.329	0.150	0.035	0.623	2.194	0.029	0.038		
HSE	− 0.255	0.034	− 0.322	− 0.189	− 7.530	< 0.001	< 0.001	1.241	
Gratitude	− 0.122	0.032	− 0.186	− 0.059	− 3.766	< 0.001	< 0.001	1.145	
Age	− 0.015	0.003	− 0.020	− 0.010	− 5.490	< 0.001	< 0.001	1.163	
Sex (female)	0.148	0.063	0.025	0.271	2.359	0.019	0.027	1.037	
Conditions	0.208	0.023	0.163	0.254	9.048	< 0.001	< 0.001	1.300	
**Effect^b^**	**β**	***SE***_β_	**95% CI β**		***z***	***p***	***Adj. p***	***VIF***	***OR***	**95% CI *OR***	
			*LL*	*UL*						*LL*	*UL*
Sleep									*LL* = −886.06, *df* = 2,351		
0|1	0.817	0.281	0.266	1.368	2.906	0.004	0.006		2.264	1.305	3.927
1|2	− 1.597	0.280	− 2.146	− 1.048	− 5.701	< 0.001	< 0.001		0.202	0.117	0.351
2|3	− 2.710	0.290	− 3.280	− 2.141	− 9.331	< 0.001	< 0.001		0.067	0.038	0.118
Gratitude	0.086	0.053	− 0.017	0.190	1.631	0.103	0.111	1.179	1.090	0.983	1.209
HSE_1_	− 0.100	0.090	− 0.276	0.077	− 1.109	0.267	0.267	1.397^c^	0.905	0.759	1.080
HSE_2_	− 0.365	0.081	− 0.522	− 0.207	− 4.527	< 0.001	< 0.001		0.694	0.593	0.813
HSE_3_	− 0.206	0.103	− 0.408	− 0.003	− 1.992	0.046	**0.055**		0.814	0.665	0.997
Stress	0.217	0.066	0.087	0.346	3.276	0.001	0.002	2.013	1.242	1.091	1.414
Anxiety	0.440	0.069	0.305	0.575	6.392	< 0.001	< 0.001	2.235	1.553	1.357	1.778
Depression	0.291	0.072	0.150	0.432	4.047	< 0.001	< 0.001	2.384	1.337	1.162	1.540
Age	0.009	0.004	0.000	0.018	2.055	0.040	**0.054**	1.236	1.009	1.000	1.018
Education	− 0.074	0.036	− 0.145	− 0.003	− 2.039	0.041	**0.054**	1.110	0.929	0.865	0.997
Conditions	0.177	0.037	0.105	0.250	4.783	< 0.001	< 0.001	1.416	1.194	1.110	1.284

Standardized coefficients are reported.

CI, confidence interval; LL, lower limit; UL, upper limit. Adj. p, Adjusted p-value (FDR correction); VIF, variance inflation factor; OR, odds ratio; HSE, health self-efficacy.

Bolded p-values indicate those that became non-significant after adjustment. 0|1: sleep disturbances threshold; the likelihood of scoring a 0 on sleep disturbances as opposed to 1−3. 1|2: sleep disturbances threshold; the likelihood of scoring a 0−1 on sleep disturbances as opposed to 2−3. 2|3: sleep disturbances threshold; the likelihood of scoring 0−2 on sleep disturbances as opposed to 3. HSE_1_: The likelihood of a one standard deviation increase in health self-efficacy being associated with higher sleep disturbances scores of 1−3 vs. 0. HSE_2_: The likelihood of a one standard deviation increase in health self-efficacy being associated with higher sleep disturbances scores of 2-3 vs. 0−1. HSE_3_: The likelihood of a one standard deviation increase in health self-efficacy being associated with a higher sleep disturbances score of 3 vs. 0−2.

Covariates: Age = participant age; Sex = participant sex; Education = participant education level; Conditions = number of chronic medical conditions.

^a^Multiple linear regression analyses.

^b^Ordinal logistic regression analysis.

^c^Variance inflation factor for health self-efficacy.

Mediation effect results for model 1 are provided in Table 4. As hypothesized, health self-efficacy as a first-order mediator, and stress (*a*_1_*d*_21_*b*_4_= −0.019, 95% CI [−0.039, −0.002], *SE* = 0.010), anxiety (*a*_1_*d*_31_*b*_5_ = −0.026, 95% CI [−0.045, −0.008], *SE* = 0.009), and depression (*a*_1_*d*_41_*b*_6_ = −0.020, 95% CI [−0.040, −0.003], *SE* = 0.009) as parallel second-order mediators, serially mediated the relation between gratitude and sleep disturbances. Additionally, specific indirect effects linking gratitude and sleep disturbances were observed for one of the three health self-efficacy pathways, HSE_2_ (*a*_1_*b*_2_ = −0.099, 95% CI [−0.166, −0.043], *SE* = 0.031), and stress (*a*_2_*b*_4_ = −0.033, 95% CI [−0.072, −0.003], *SE* = 0.018) and depression (*a*_4_*b*_6_ = −0.036, 95% CI [−0.078, −0.004], *SE* = 0.019). Overall, the total indirect effect (β = −0.347, 95% CI [−0.530, −0.066], *SE* = 0.105) and the total effect of gratitude on sleep disturbances (*c* = −0.261, 95% CI [−0.478, −0.030], *SE* = 0.112) were significant, while the direct effect was non-significant (*c'* = 0.086, 95% CI [−0.066, 0.235], *SE* = 0.078), indicating mediation. After adjusting the confidence intervals to 0.97 to account for false coverage rate correction, the conclusions regarding all indirect effects remained consistent.

**Table 4:** Indirect effects of health self-efficacy and psychological distress on the relation between gratitude and sleep disturbances (Model 1).

**Effect**		**β**	***SE***_β_	**95% CI** β	
				***LL***	***UL***
Gratitude → self-efficacy_1_ → sleep	*a_1_ b_1_*	−0.027	0.028	−0.084	0.027
Gratitude → self-efficacy_2_ → sleep	*a_1_ b_2_*	−0.099	0.031	−0.166	−0.043
Gratitude → self-efficacy_3_ → sleep	*a_1_ b_3_*	−0.056	0.046	−0.137	0.064
Gratitude → stress → sleep	*a_2_ b_4_*	−0.033	0.018	−0.072	−0.003
Gratitude → anxiety → sleep	*a_3_ b_5_*	−0.030	0.019	−0.072	0.003
Gratitude → depression → sleep	*a_4_ b_6_*	−0.036	0.019	−0.078	−0.004
Gratitude → self-efficacy → stress → sleep	*a_1_ d_21_ b_4_*	−0.019	0.010	−0.039	−0.002
Gratitude → self-efficacy → anxiety → sleep	*a_1_ d_31_ b_5_*	−0.026	0.009	−0.045	−0.008
Gratitude → self-efficacy → depression → sleep	*a_1_ d_41_ b_6_*	−0.020	0.009	−0.040	−0.003
Total	*c*	−0.261	0.112	−0.478	−0.030
Total indirect		−0.347	0.105	−0.530	−0.066
Direct	*c*′	0.086	0.078	−0.066	0.235

Covariates include age, sex, education level, and number of chronic diseases. Standardized coefficients are reported.

CI, confidence interval; LL, lower limit; UL, upper limit. Self-efficacy = health self-efficacy.

Self-efficacy_1_: Specific indirect effect of gratitude related to sleep disturbances scores of 1–3 vs. 0, via health self-efficacy. Self-efficacy_2_: Specific indirect effect of gratitude related to sleep disturbances scores of 2–3 vs. 0–1, via health self-efficacy. Self-efficacy_3_: Specific indirect effect of gratitude related to sleep disturbances scores of 3 vs. 0–2, via health self-efficacy. All effects remained consistent after adjusting the CIs for the FCR at 0.97.

#### 3.2.2 Model 2

Regarding model 2, the ordinal logistic regression model fit the data significantly better than the multinomial model ($\chi_{\left(10\right)}^{2}$ = 98.897, *p* ≤ 0.001) and was more parsimonious. However, the proportional odds assumption was violated for health behaviors ($\chi_{\left(2\right)}^{2}$ = 6.558, *p* = 0.038). Therefore, a partial proportional odds model including three regression coefficients for health behaviors was used.

**Figure 3:**
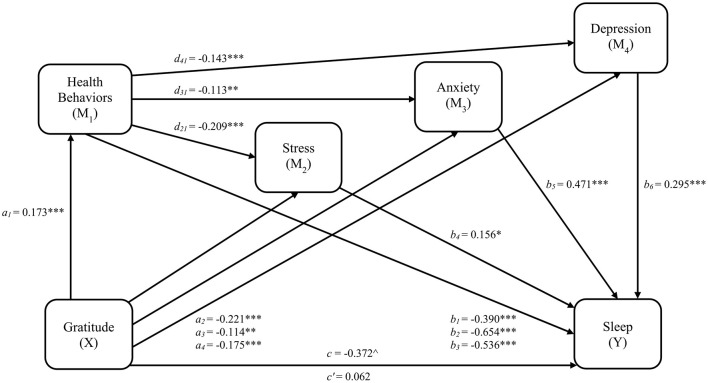
Serial indirect effects Model 2 for gratitude, health behaviors, psychological distress, and sleep disturbances. *a*_1_*, a*_2_*, a*_3_*, a*_4_ = regression coefficients for gratitude to health behaviors, stress, anxiety, and depression, respectively. *b*_1_*, b*_2_*, b*_3_ = regression coefficients for health behaviors to each threshold of sleep disturbances scores (0|1, 1|2, and 2|3, respectively). *b*_4_*, b*_5_*, b*_6_ = regression coefficients for stress, anxiety, and depression, respectively, to sleep disturbances. *d*_21_*, d*_31_*, d*_41_ = regression coefficients for health behaviors to stress, anxiety, and depression, respectively; *c* = total effect (gratitude related to sleep disturbances); *c*' = direct effect (gratitude related to sleep disturbances accounting for health behaviors and psychological distress). *p < 0.05; **p < 0.01; ***p < 0.001. *p*-values are FDR-adjusted. ^∧^Significance indicated by 95% CI that does not cross zero.

Linear and ordinal logistic regression results and fit statistics for each pathway, including significant covariates identified through backward stepwise selection, are provided in Table 5. Variance inflation factors are also provided in Table 5, and no predictors exceed the multicollinearity cutoff. Regression results excluding covariate effects are illustrated in Figure 3. Gratitude was related to better health behavior engagement (β = 0.173, 95% CI [0.106, 0.239], *SE* = 0.034, *t* = 5.081, *p*_*adjusted*_ < 0.001) which was, in turn, related to lower stress (β = −0.209, 95% CI [−0.274, −0.144], *SE* = 0.033, *t* = −6.290, *p*_*adjusted*_ < 0.001), anxiety (β = −0.113, 95% CI [−0.180, −0.046], *SE* = 0.034, *t* = −3.314, *p*_*adjusted*_ = 0.002), and depression (β = −0.143, 95% CI [−0.207, −0.079], *SE* = 0.033, *t* = −4.393, *p*_*adjusted*_ < 0.001). A one standard deviation increase in stress was associated with a 17% greater likelihood of endorsing a higher frequency of sleep disturbances (*OR* = 1.168, 95% CI [1.026, 1.330], *p*_*adjusted*_ = 0.024), while equivalent increases in anxiety (*OR* = 1.602, 95% CI [1.394, 1.840], *p*_*adjusted*_ < 0.001) and depression (*OR* = 1.344, 95% CI [1.166, 1.549], *p*_*adjusted*_ < 0.001) were associated with 60% and 34% greater likelihood of sleep problem frequency, respectively.

**Table 5:** Regression coefficients of serial indirect effects Model 2 for gratitude, health behaviors, psychological distress, and sleep disturbances, with covariates.

**Effect^a^**	**β**	***SE***_β_	**95% CI β**		***t***	***p***	***Adj. p***	***VIF***	**Fit statistics**
			*LL*	*UL*					
HB									*R*^2^ = 0.126;*R*^2^_adjusted_ = 0.122*F*_(4,817)_ = 29.38,*p* < 0.001
(Intercept)	− 1.481	0.187	− 1.848	− 1.114	− 7.922	< 0.001	< 0.001		
Gratitude	0.173	0.034	0.106	0.239	5.081	< 0.001	< 0.001	1.051	
Age	0.026	0.003	0.021	0.032	8.970	< 0.001	< 0.001	1.158	
Education	0.074	0.025	0.025	0.122	2.995	0.003	0.004	1.075	
Conditions	− 0.097	0.024	− 0.144	− 0.050	− 4.028	< 0.001	< 0.001	1.179	
Stress									*R*^2^ = 0.211;*R*^2^_adjusted_ = 0.206*F*_(5,820)_ = 43.8, *p* < 0.001
(Intercept)	0.082	0.162	− 0.235	0.400	0.508	0.612	0.629		
HB	− 0.209	0.033	− 0.274	− 0.144	− 6.290	< 0.001	< 0.001	1.120	
Gratitude	− 0.221	0.033	− 0.286	− 0.157	− 6.729	< 0.001	< 0.001	1.082	
Age	− 0.009	0.003	− 0.015	− 0.003	− 3.095	0.002	0.003	1.269	
Sex (female)	0.113	0.065	− 0.015	0.242	1.733	0.083	0.104	1.043	
Conditions	0.187	0.023	0.142	0.232	8.180	< 0.001	< 0.001	1.205	
Anxiety									*R*^2^ = 0.148;*R*^2^_adjusted_ = 0.142*F*_(5,826)_ = 28.58,*p* < 0.001
(Intercept)	− 0.007	0.166	− 0.332	0.318	− 0.041	0.967	0.967		
HB	− 0.113	0.034	− 0.180	− 0.046	− 3.314	< 0.001	0.002	1.123	
Gratitude	− 0.114	0.034	− 0.181	− 0.048	− 3.385	< 0.001	0.001	1.084	
Age	− 0.010	0.003	− 0.016	− 0.004	− 3.269	0.001	0.002	1.262	
Sex (female)	0.257	0.067	0.125	0.389	3.827	< 0.001	< 0.001	1.041	
Conditions	0.207	0.023	0.161	0.253	8.844	< 0.001	< 0.001	1.202	
Depression									*R*^2^ = 0.217;*R*^2^_adjusted_ = 0.213*F*_(5,826)_ = 45.88,*p* < 0.001
(Intercept)	0.160	0.157	− 0.149	0.469	1.018	0.309	0.348		
HB	− 0.143	0.033	− 0.207	− 0.079	− 4.393	< 0.001	< 0.001	1.120	
Gratitude	− 0.175	0.032	− 0.238	− 0.112	− 5.433	< 0.001	< 0.001	1.084	
Age	− 0.013	0.003	− 0.019	− 0.007	− 4.562	< 0.001	< 0.001	1.259	
Sex (female)	0.159	0.064	0.033	0.284	2.480	0.013	0.017	1.041	
Conditions	0.242	0.022	0.198	0.286	10.821	< 0.001	< 0.001	1.201	
**Effect^b^**	**β**	***SE***_β_	**95% CI β**		***z***	***p***	***Adj. p***	***VIF***	***OR***	**95% CI** *OR*	
			*LL*	*UL*						*LL*	*UL*
Sleep									*LL* = −888.96, *df* = 2,417		
0|1	− 0.257	0.256	− 0.759	0.244	− 1.005	0.315	0.315		0.773	0.468	1.277
1|2	− 2.748	0.265	− 3.267	− 2.229	− 10.374	< 0.001	< 0.001		0.064	0.038	0.108
2|3	− 3.931	0.280	− 4.480	− 3.382	− 14.024	< 0.001	< 0.001		0.020	0.011	0.034
Gratitude	0.062	0.053	− 0.041	0.166	1.184	0.236	0.256	1.153	1.064	0.960	1.180
HB_1_	− 0.390	0.093	− 0.572	− 0.208	− 4.201	< 0.001	< 0.001	1.175^c^	0.677	0.564	0.812
HB_2_	− 0.654	0.077	− 0.804	− 0.503	− 8.499	< 0.001	< 0.001		0.520	0.447	0.605
HB_3_	− 0.536	0.097	− 0.726	− 0.345	− 5.514	< 0.001	< 0.001		0.585	0.484	0.708
Stress	0.156	0.066	0.026	0.285	2.350	0.019	0.024	1.975	1.168	1.026	1.330
Anxiety	0.471	0.071	0.332	0.610	6.650	< 0.001	< 0.001	2.269	1.602	1.394	1.840
Depression	0.295	0.073	0.153	0.438	4.070	< 0.001	< 0.001	2.417	1.344	1.166	1.549
Age	0.021	0.005	0.012	0.030	4.516	< 0.001	< 0.001	1.306	1.021	1.012	1.031
Sex (female)	0.216	0.101	0.018	0.414	2.135	0.033	0.039	1.069	1.241	1.018	1.513
Conditions	0.236	0.037	0.163	0.309	6.356	< 0.001	< 0.001	1.394	1.266	1.177	1.361

Standardized coefficients are reported.

CI, confidence interval; LL, lower limit; UL, upper limit; Adj. p, Adjusted p-value (FDR correction); VIF, variance inflation factor; OR, odds ratio; HB, health behaviors.

0|1: sleep disturbances threshold; the likelihood of scoring a 0 on sleep disturbances as opposed to 1−3. 1|2: sleep disturbances threshold; the likelihood of scoring a 0−1 on sleep disturbances as opposed to 2−3. 2|3: sleep disturbances threshold; the likelihood of scoring 0−2 on sleep disturbances as opposed to 3. HB_1_: The likelihood of a one standard deviation increase in health behaviors being associated with higher sleep disturbances scores of 1−3 vs. 0. HB_2_: The likelihood of a one standard deviation increase in health behaviors being associated with higher sleep disturbances scores of 2−3 vs. 0−1. HB_3_: The likelihood of a one standard deviation increase in health behaviors being associated with a higher sleep disturbances score of 3 vs. 0−2. Covariates: Age = participant age; Sex = participant sex; Education = participant education level; Conditions = number of chronic medical conditions.

^a^Multiple linear regression analyses.

^b^Ordinal logistic regression analysis.

^c^Variance inflation factor for health behaviors.

Mediation effect results for model 2 are provided in Table 6. Despite significant regression results, the multivariate hypothesis for model 2 was only partially supported. Serial mediation was observed through health behaviors as a first-order mediator, and anxiety (*a*_1_*d*_31_*b*_5_= −0.009, 95% CI [−0.019, −0.002], *SE* = 0.004) and depression (*a*_1_*d*_41_*b*_6_= −0.007, 95% CI [−0.016, −0.001], *SE* = 0.004), but not stress, as parallel second-order mediators. Additionally, specific indirect effects linking gratitude and sleep disturbances were observed for all three health behavior pathways, health behaviors_1_ (*a*_1_*b*_1_= −0.067, 95% CI [−0.120, −0.026], *SE* = 0.024), health behaviors_2_ (*a*_1_*b*_2_= −0.113, 95% CI [−0.180, −0.059], *SE* = 0.030), health behaviors_3_ (*a*_1_*b*_3_= −0.092, 95% CI [−0.158, −0.040], *SE* = 0.032), as well as anxiety (*a*_3_*b*_5_= −0.054, 95% CI [−0.104, −0.015], *SE* = 0.022) and depression (*a*_4_*b*_6_= −0.052, 95% CI [−0.103, −0.011], *SE* = 0.024). Overall, the total indirect effect (β = −0.435, 95% CI [−0.624, −0.268], *SE* = 0.093) and the total effect of gratitude on sleep disturbances (*c* = −0.372, 95% CI [−0.607, −0.146], *SE* = 0.115) were significant, while the direct effect was non-significant (*c*' = 0.062, 95% CI [−0.090, 0.209], *SE* = 0.076), indicating mediation. After adjusting the confidence intervals to 0.97 to account for false coverage rate correction, the conclusions regarding all indirect effects remained consistent.

**Table 6:** Indirect effects of health behaviors and psychological distress on the relation between gratitude and sleep disturbances (Model 2).

**Effect**		**β**	***SE***_β_	**95% CI** β	
				***LL***	***UL***
Gratitude → health behaviors_1_ → sleep	*a_1_ b_1_*	−0.067	0.024	−0.120	−0.026
Gratitude → health behaviors_2_ → sleep	*a_1_ b_2_*	−0.113	0.030	−0.180	−0.059
Gratitude → health behaviors_3_ → sleep	*a_1_ b_3_*	−0.092	0.032	−0.158	−0.040
Gratitude → stress → sleep	*a_2_ b_4_*	−0.034	0.022	−0.081	0.007
Gratitude → anxiety → sleep	*a_3_ b_5_*	−0.054	0.022	−0.104	−0.015
Gratitude → depression → sleep	*a_4_ b_6_*	−0.052	0.024	−0.103	−0.011
Gratitude → health behaviors → stress → sleep	*a_1_ d_21_ b_4_*	−0.006	0.004	−0.014	0.001
Gratitude → health behaviors → anxiety → sleep	*a_1_ d_31_ b_5_*	−0.009	0.004	−0.019	−0.002
Gratitude → health behaviors → depression → sleep	*a_1_ d_41_ b_6_*	−0.007	0.004	−0.016	−0.001
Total	*c*	−0.372	0.115	−0.607	−0.146
Total indirect		−0.435	0.093	−0.624	−0.268
Direct	*c*′	0.062	0.076	−0.090	0.209

Covariates include age, sex, education level, and number of chronic diseases. Standardized coefficients are reported.

CI, confidence interval; LL, lower limit; UL, upper limit.

Health behaviors_1_: Specific indirect effect of gratitude related to sleep disturbances scores of 1–3 vs. 0, via health behaviors. Health behaviors_2_: Specific indirect effect of gratitude related to sleep disturbances scores of 2–3 vs. 0–1, via health behaviors. Health behaviors_3_: Specific indirect effect of gratitude related to sleep disturbances scores of 3 vs. 0–2, via health behaviors. All effects remained consistent after adjusting the CIs for the FCR at 0.97.

#### 3.2.3 Model 3

Regarding model 3, the ordinal logistic regression model fit the data significantly better than the multinomial model ($\chi_{\left(12\right)}^{2}$ = 549.04, *p* ≤ 0.001) and was more parsimonious. However, the proportional odds assumption was violated for both health self-efficacy and health behaviors (HSE: $\chi_{\left(2\right)}^{2}$ = 6.990, *p* = 0.030; health behaviors: $\chi_{\left(2\right)}^{2}$ = 6.712, *p* = 0.035; both: $\chi_{\left(4\right)}^{2}$ = 10.397, *p* = 0.034). As all three models fit significantly better than the parallel model, the partial proportional odds model containing multiple slopes for both health self-efficacy and health behaviors was then compared to models containing multiple slopes for either health self-efficacy or health behaviors, to determine the most parsimonious model. The model including multiple slopes for both health self-efficacy and health behaviors did not fit the data significantly better than the health self-efficacy-only ($\chi_{\left(2\right)}^{2}$ = 3.407, *p* = 0.182) or health behaviors-only ($\chi_{\left(2\right)}^{2}$ = 3.685, *p* = 0.158) models. Next, the health self-efficacy-only and health behaviors-only models were compared. The health self-efficacy-only model fit better than the health behaviors-only model ($\chi_{\left(0\right)}^{2}$ = 0.278, *p* < 0.001); thus, the health self-efficacy-only partial proportional odds model was used.

**Figure 4:**
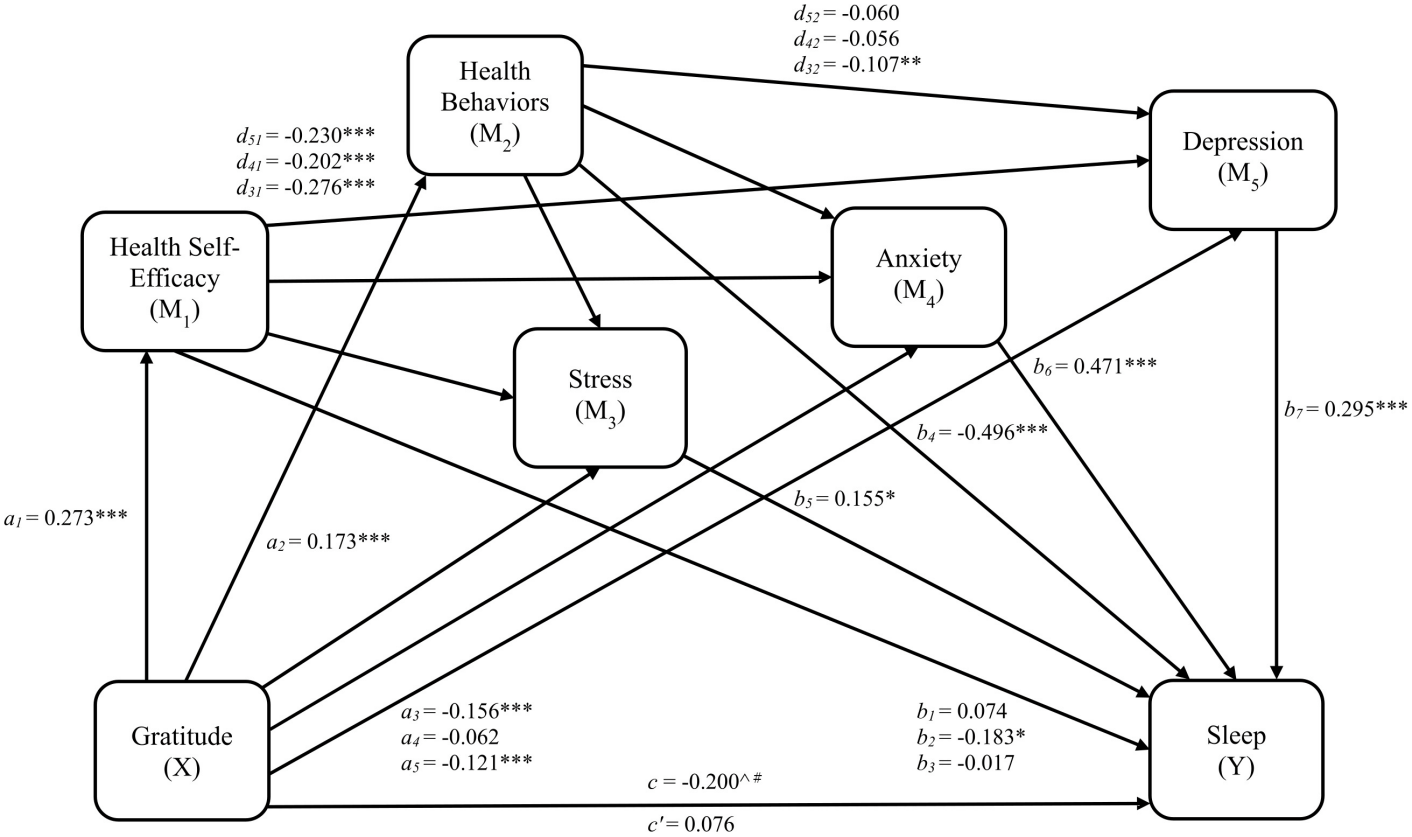
Serial indirect effects Model 3 for gratitude, health self-efficacy, health behaviors, psychological distress, and sleep disturbances. *a*_1_*, a*_2_*, a*_3_*, a*_4_*, a*_5_ = regression coefficients for gratitude to health self-efficacy, health behaviors, stress, anxiety, and depression, respectively. *b*_1_*, b*_2_*, b*_3_ = regression coefficients for health self-efficacy to each threshold of sleep disturbances scores (0|1, 1|2, and 2|3, respectively). *b*_4_*, b*_5_*, b*_6_, *b*_7_ = regression coefficients for health behaviors, stress, anxiety, and depression, respectively, to sleep disturbances. *d*_31_*, d*_41_*, d*_51_ = regression coefficients for health self-efficacy to stress, anxiety, and depression, respectively; *d*_32_*, d*_42_*, d*_52_ = regression coefficients for health behaviors to stress, anxiety, and depression, respectively; *c* = total effect (gratitude related to sleep disturbances); *c*' = direct effect (gratitude related to sleep disturbances accounting for health self-efficacy, health behaviors, and psychological distress).*p < 0.05; **p < 0.01; ***p < 0.001. *p*-values are FDR-adjusted. ^∧^Significance indicated by 95% CI that does not cross zero. ^#^Indicates effect became non-significant after adjusting confidence interval to 97%.

Linear and ordinal logistic regression results and fit statistics for each pathway, including significant covariates identified through backward stepwise selection, are provided in Table 7. Variance inflation factors are also provided in Table 7, and no predictors exceed the multicollinearity cutoff. Regression results excluding covariate effects are illustrated in Figure 4. Gratitude was related to better health self-efficacy (β = 0.273, 95% CI [0.209, 0.337], *SE* = 0.032, *t* = 8.412, *p*_*adjusted*_ < 0.001) which was, in turn, related to lower stress (β = −0.276, 95% CI [−0.350, −0.203], *SE* = 0.038, *t* = −7.352, *p*_*adjusted*_ < 0.001), anxiety (β = −0.202, 95% CI [−0.280, −0.123], *SE* = 0.040, *t* = −5.064, *p*_*adjusted*_ < 0.001), and depression (β = −0.230, 95% CI [−0.303, −0.157], *SE* = 0.037, *t* = −6.169, *p*_*adjusted*_ < 0.001). Gratitude was also related to better health behavior engagement (β = 0.173, 95% CI [0.106, 0.239], *SE* = 0.034, *t* = 5.081, *p*_*adjusted*_ < 0.001) which was, in turn, related to lower stress (β = −0.107, 95% CI [−0.177, −0.037], *SE* = 0.036, *t* = −3.001, *p*_*adjusted*_ = 0.005), but not anxiety and depression. A one standard deviation increase in stress was associated with a 17% greater likelihood of endorsing a higher frequency of sleep disturbances (*OR* = 1.168, 95% CI [1.023, 1.333], *p*_*adjusted*_ = 0.038), while equivalent increases in anxiety (*OR* = 1.602, 95% CI [1.394, 1.840], *p*_*adjusted*_ < 0.001) and depression (*OR* = 1.343, 95% CI [1.163, 1.551], *p*_*adjusted*_ < 0.001) were associated with 60% and 34% greater likelihood of sleep problem frequency, respectively.

**Table 7:** Regression coefficients of serial indirect effects Model 3 for gratitude, health self-efficacy, health behaviors, psychological distress, and sleep disturbances, with covariates.

**Effect^a^**	**β**	***SE***_β_	**95% CI β**		***t***	***p***	***Adj. p***	***VIF***	**Fit statistics**
			***LL***	***UL***					
HSE									*R*^2^ = 0.201;*R*^2^_adjusted_ = 0.197*F*_(4,807)_ = 50.74,*p* < 0.001
(Intercept)	− 0.361	0.179	− 0.713	− 0.010	− 2.018	0.044	**0.065**		
Gratitude	0.273	0.032	0.209	0.337	8.412	< 0.001	< 0.001	1.052	
Age	0.008	0.003	0.003	0.014	2.938	0.003	0.006	1.158	
Education	0.077	0.023	0.031	0.123	3.300	0.001	0.002	1.074	
Conditions	− 0.196	0.023	− 0.242	− 0.151	− 8.488	< 0.001	< 0.001	1.181	
HB									*R*^2^ = 0.126;*R*^2^_adjusted_ = 0.122*F*_(4,817)_ = 29.38,*p* < 0.001
(Intercept)	− 1.481	0.187	− 1.848	− 1.114	− 7.922	< 0.001	< 0.001		
Gratitude	0.173	0.034	0.106	0.239	5.081	< 0.001	< 0.001	1.051	
Age	0.026	0.003	0.021	0.032	8.970	< 0.001	< 0.001	1.158	
Education	0.074	0.025	0.025	0.122	2.995	0.003	0.005	1.075	
Conditions	− 0.097	0.024	− 0.144	− 0.050	− 4.028	< 0.001	< 0.001	1.179	
Stress									*R*^2^ = 0.267;*R*^2^_adjusted_ = 0.261*F*_(6,808)_ = 49.01,*p* < 0.001
(Intercept)	0.212	0.157 −0.097	0.522	1.349	0.178	0.199			
HSE	− 0.276	0.038	− 0.350	− 0.203	− 7.352	< 0.001	< 0.001	1.515	
HB	− 0.107	0.036	− 0.177	− 0.037	− 3.001	0.003	0.005	1.369	
Gratitude	− 0.156	0.033	− 0.220	− 0.092	− 4.779	< 0.001	< 0.001	1.146	
Age	− 0.010	0.003	− 0.016	− 0.005	− 3.611	< 0.001	< 0.001	1.269	
Sex (female)	0.118	0.063	− 0.006	0.243	1.873	0.061	0.086	1.041	
Conditions	0.147	0.023	0.101	0.192	6.329	< 0.001	< 0.001	1.303	
Anxiety									*R*^2^ = 0.171;*R*^2^_adjusted_ = 0.164*F*_(7,795)_ = 23.4, *p* < 0.001
(Intercept)	− 0.185	0.202	− 0.581	0.212 −0.914	0.361	0.386			
HSE	− 0.202	0.040	− 0.280	− 0.123	− 5.064	< 0.001	< 0.001	1.518	
HB	− 0.056	0.038	− 0.130	0.018	− 1.481	0.139	0.160	1.389	
Gratitude	− 0.062	0.035	− 0.130	0.006	− 1.780	0.075	0.102	1.146	
Age	− 0.009	0.003	− 0.015	− 0.003	− 2.883	0.004	0.007	1.300	
Sex (female)	0.278	0.067	0.147	0.410	4.148	< 0.001	< 0.001	1.042	
Education	0.048	0.024	0.001	0.096	1.999	0.046	**0.066**	1.096	
Conditions	0.171	0.025	0.123	0.220	6.966	< 0.001	< 0.001	1.292	
Depression									*R*^2^ = 0.256;*R*^2^_adjusted_ = 0.251*F*_(6,813)_ = 46.72,*p* < 0.001
(Intercept)	0.241	0.155	− 0.063	0.546	1.555	0.120	0.142		
HSE	− 0.230	0.037	− 0.303	− 0.157	− 6.169	< 0.001	< 0.001	1.512	
HB	− 0.060	0.035	− 0.129	0.009	− 1.720	0.086	0.113	1.365	
Gratitude	− 0.121	0.032	− 0.185	− 0.058	− 3.756	< 0.001	< 0.001	1.146	
Age	− 0.013	0.003	− 0.019	− 0.008	− 4.756	< 0.001	< 0.001	1.259	
Sex (female)	0.157	0.063	0.034	0.280	2.510	0.012	0.019	1.038	
Conditions	0.206	0.023	0.161	0.251	9.006	< 0.001	< 0.001	1.300	
**Effect^b^**	**β**	***SE***_β_	**95% CI β**		***z***	***p***	***Adj. p***	***VIF***	***OR***	**95% CI OR**	
			*LL*	*UL*						*LL*	*UL*
Sleep									*LL* = -879.75, *df* = 2,386		
0|1	− 0.182	0.258	− 0.687	0.324	− 0.704	0.482	0.519		0.834	0.503	1.383
1|2	− 2.660	0.266	− 3.181	− 2.139	− 10.006	< 0.001	< 0.001		0.070	0.042	0.118
2|3	− 3.837	0.281	− 4.388	− 3.286	− 13.653	< 0.001	< 0.001		0.022	0.012	0.037
Gratitude	0.076	0.054	− 0.029	0.182	1.413	0.158	0.200	1.190	1.079	0.971	1.199
HSE_1_	0.074	0.094	− 0.111	0.259	0.786	0.432	0.504	1.633^c^	1.077	0.895	1.296
HSE_2_	− 0.183	0.086	− 0.351	− 0.015	− 2.140	0.032	0.045		0.832	0.704	0.985
HSE_3_	− 0.017	0.109	− 0.230	0.196	− 0.157	0.875	0.875		0.983	0.794	1.217
HB	− 0.496	0.057	− 0.608	− 0.384	− 8.684	< 0.001	< 0.001	1.377	0.609	0.545	0.681
Stress	0.155	0.068	0.023	0.288	2.295	0.022	0.038	2.044	1.168	1.023	1.333
Anxiety	0.471	0.071	0.332	0.610	6.638	< 0.001	< 0.001	2.281	1.602	1.394	1.840
Depression	0.295	0.074	0.151	0.439	4.013	< 0.001	< 0.001	2.435	1.343	1.163	1.551
Age	0.020	0.005	0.011	0.030	4.344	< 0.001	< 0.001	1.310	1.021	1.011	1.030
Sex (female)	0.226	0.101	0.027	0.425	2.227	0.026	0.040	1.065	1.253	1.027	1.529
Conditions	0.207	0.038	0.132	0.282	5.406	< 0.001	< 0.001	1.448	1.230	1.141	1.325

Standardized coefficients are reported.

CI, confidence interval; LL, lower limit; UL, upper limit. Adj. p, Adjusted p-value (FDR correction); VIF, variance inflation factor; OR, odds ratio; HSE, health self-efficacy; HB, health behaviors.

Bolded p-values indicate those that became non-significant after adjustment. 0|1: sleep disturbances threshold; the likelihood of scoring a 0 on sleep disturbances as opposed to 1−3. 1|2: sleep disturbances threshold; the likelihood of scoring a 0−1 on sleep disturbances as opposed to 2−3. 2|3: sleep disturbances threshold; the likelihood of scoring 0−2 on sleep disturbances as opposed to 3. HSE_1_: The likelihood of a one standard deviation increase in health self-efficacy being associated with higher sleep disturbances scores of 1−3 vs. 0. HSE_2_: The likelihood of a one standard deviation increase in health self-efficacy being associated with higher sleep disturbances scores of 2−3 vs. 0−1. HSE_3_: The likelihood of a one standard deviation increase in health self-efficacy being associated with a higher sleep disturbances score of 3 vs. 0−2.

Covariates: Age = participant age; Sex = participant sex; Education = participant education level; Conditions = number of chronic medical conditions.

^a^Multiple linear regression analyses.

^b^Ordinal logistic regression analysis.

^c^Variance inflation factor for health self-efficacy.

Mediation effects results for model 3 are provided in Table 8. The multivariate hypothesis for model 3 was only partially supported. No serial mediation was found when health behaviors was included as a first-order mediator and, as a result, health self-efficacy and health behaviors did not function as parallel first-order mediators. Only health self-efficacy and anxiety (*a*_1_*d*_41_*b*_6_ = −0.026, 95% CI [−0.046, −0.009], *SE* = 0.009), and health self-efficacy and depression (*a*_1_*d*_51_*b*_7_ = −0.019, 95% CI [−0.037, −0.003], *SE* = 0.009), serially mediated the relation between gratitude and sleep disturbances. Specific indirect effects linking gratitude and sleep disturbances were observed for health behaviors (*a*_2_*b*_4_ = −0.086, 95% CI [−0.140, −0.030], *SE* = 0.026) and depression (*a*_5_*b*_7_= −0.036, 95% CI [−0.079, −0.005], *SE* = 0.019). Despite serial mediation occurring in only two of six potential paths, the sum of all indirect effects of gratitude on sleep disturbances via health self-efficacy, health behaviors, and psychological distress was significant (β = −0.276, 95% CI [−0.451, −0.053], *SE* = 0.093), as was the total effect (*c* = −0.200, 95% CI [−0.412, −0.001], *SE* = 0.105). The direct effect was non-significant (*c*' = 0.076, 95% CI [−0.078, 0.227], *SE* = 0.079), indicating mediation. After adjusting the confidence intervals to 0.97 to account for false coverage rate correction, the conclusions regarding all indirect effects remained consistent. However, the total effect of gratitude on sleep became non-significant after adjustment, indicating a suppression effect in which the positive direct and negative indirect effects cancel each other out (MacKinnon, 2000), and likely reflecting the lack of serial indirect effects for health behaviors.

**Table 8:** Indirect effects of health self-efficacy, health behaviors, and psychological distress on the relation between gratitude and sleep disturbances (Model 3).

**Effect**		**β**	***SE***_β_	**95% CI** β	
				***LL***	***UL***
Gratitude → self-efficacy_1_ → sleep	*a_1_ b_1_*	0.020	0.030	−0.037	0.079
Gratitude → self-efficacy_2_ → sleep	*a_1_ b_2_*	−0.050	0.030	−0.113	0.009
Gratitude → self-efficacy_3_ → sleep	*a_1_ b_3_*	−0.005	0.041	−0.082	0.081
Gratitude → health behaviors → sleep	*a_2_ b_4_*	−0.086	0.026	−0.140	−0.030
Gratitude → stress → sleep	*a_3_ b_5_*	−0.024	0.017	−0.061	0.006
Gratitude → anxiety → sleep	*a_4_ b_6_*	−0.029	0.020	−0.073	0.006
Gratitude → depression → sleep	*a_5_ b_7_*	−0.036	0.019	−0.079	−0.005
Gratitude → self-efficacy → stress → sleep	*a_1_ d_31_ b_5_*	−0.012	0.008	−0.029	0.003
Gratitude → self-efficacy → anxiety → sleep	*a_1_ d_41_ b_6_*	−0.026	0.009	−0.046	−0.009
Gratitude → self-efficacy → depression → sleep	*a_1_ d_51_ b_7_*	−0.019	0.009	−0.037	−0.003
Gratitude → health behaviors → stress → sleep	*a_2_ d_32_ b_5_*	−0.003	0.002	−0.008	0.001
Gratitude → health behaviors → anxiety → sleep	*a_2_ d_42_ b_6_*	−0.005	0.004	−0.013	0.002
Gratitude → health behaviors → depression → sleep	*a_2_ d_52_ b_7_*	−0.003	0.003	−0.009	0.001
Total	*c*	−0.200	0.105	−0.412	−**0.001**
Total indirect		−0.276	0.093	−0.451	−0.053
Direct	*c*′	0.076	0.079	−0.078	0.227

Covariates include age, sex, education level, and number of chronic diseases. Standardized coefficients are reported.

CI, confidence interval; LL, lower limit; UL, upper limit; Self-efficacy, health self-efficacy.

Self-efficacy_1_: Specific indirect effect of gratitude related to sleep disturbances scores of 1–3 vs. 0, via health self-efficacy. Self-efficacy_2_: Specific indirect effect of gratitude related to sleep disturbances scores of 2–3 vs. 0-1, via health self-efficacy. Self-efficacy_3_: Specific indirect effect of gratitude related to sleep disturbances scores of 3 vs. 0–2, via health self-efficacy. All effects remained consistent after adjusting the CIs for the FCR at 0.97 except for bolded CI, which became positive, indicating a non-significant total effect.

Finally, the results of our secondary SEM analyses provided additional support for our models, with modified models (i.e., dichotomized outcome, creation of psychological distress latent variable) exhibiting good fit and comparable patterns of direct and indirect effects. Please refer to Supplementary material for detailed results.

## 4 Discussion

In a sample of German primary care patients, we examined the potential serial mediating effects of health self-efficacy (HSE) and health behaviors as first-order mediators, and stress, anxiety, and depression as second-order mediators, on the relation between gratitude and sleep disturbances. Consistent with our theoretical model, in model 1, the serial mediation hypothesis was supported; gratitude was related to greater health self-efficacy and, in turn, to less stress, anxiety, and depression in parallel, with subsequent diminished sleep disturbances. However, in model 2, although gratitude was related to health behaviors, and health behaviors were related to stress, anxiety, and depression, the hypothesis was only partially supported, in that serial mediation was observed via health behaviors and anxiety and depression, but not via health behaviors and stress. In model 3, health self-efficacy and health behaviors did not function as parallel first-order mediators as hypothesized. Instead, serial mediation emerged in only two of six potential paths, through health self-efficacy and anxiety, and health self-efficacy and depression.

Support for our theoretical model arises from existing research outlining the benefits of gratitude for health self-efficacy and health behaviors. Gratitude is a pleasant emotion that, when evoked, facilitates the development of additional positive emotions (i.e., positive affect hypothesis; Wood et al., 2010), and the building of resources to be called upon during stressful times (i.e., broaden-and-build theory, Fredrickson, 2004; Wood et al., 2010), thereby contributing to health and wellbeing. Grateful individuals employ health-promoting behaviors as a positive coping strategy and are less likely to engage in maladaptive health behaviors, such as substance use, according to the coping hypothesis (Wood et al., 2007). Further, according to the cognitive framework, gratitude stimulates a positive perception of one's abilities, strengths, and circumstances, engagement in adaptive emotion regulation strategies, and a tendency to focus on and recall encouraging experiences which, in turn, enhances resilience against stress and improves psychological and physical wellbeing (Alkozei et al., 2018). Bandura's (1989) social cognitive theory describes self-efficacy as the belief in one's ability to control life circumstances, and health self-efficacy specifically refers to belief in ability to perform necessary behaviors to attain health goals (Sirois, 2007). Self-efficacy influences health behaviors through goals, perceptions of barriers and facilitators, and outcome expectations (Bandura, 2004). Experience with prior successes helps bolster belief in capability to exert control despite setbacks (Bandura, 1977, 2004); thus, gratitude's tendency to promote a positive perspective of capabilities and experiences may improve sense of control over health (Swain et al., 2020).

Gratitude can foster self-efficacy and mitigate psychological distress and sleep problems, consistent with the significant serial indirect effects findings of models 1 and 3. For example, among first-year U.S. undergraduates with high, but not low, gratitude levels, self-efficacy for COVID-19 safety predicted less depression after 2 months (Ang et al., 2022). Further, gratitude interventions increased self-efficacy and reduced perceptions of stress in German young adults (Lorenz et al., 2022), improved pain self-efficacy and pain-related anxiety in individuals with arthritis (Swain et al., 2020), and improved environmental mastery (i.e., perceived ability to effectively change surrounding circumstances and events; Ryff and Keyes, 1995), perceived stress, anxiety, depression, and insomnia in adults (Czyżowska and Gurba, 2022). Finally, in patients with asymptomatic heart failure, gratitude was related to greater self-efficacy to preserve heart function, less depression, better sleep, and lower levels of inflammatory markers (Mills et al., 2015).

Additionally, consistent with model 2's findings, engagement in adaptive health behaviors can positively impact psychological health and sleep (Su et al., 2021), and numerous studies offer support for gratitude's salubrious influence on these associations. In a global ecological momentary assessment study of adults (*N* = 4,825), greater gratitude was associated with increased exercise, lower blood pressure, heart rate, and stress, more daily positive expectations and reflections, and better sleep quality (Newman et al., 2021). Similarly, after a gratitude diary intervention, women reporting emotional distress and moderate sleep disturbances experienced more positive emotions, reduced blood pressure, and better sleep quality (Jackowska et al., 2016). In a large online survey of 59,985 respondents from ~160 countries, gratitude was associated with increased likelihood of engaging in healthy eating, social activities, self-care, and exercise, and reduced risk for smoking, depression, anxiety, poor physical health, and insufficient sleep (Weziak-Bialowolska et al., 2023). Finally, in a large multinational online survey of pregnant women, participants endorsing greater gratitude reported less detrimental impact of COVID-19 on diet, fitness, and sleep, and did not have anxiety or depression (Choi et al., 2022).

Importantly, it may be necessary to improve self-efficacy to affect positive changes in health behaviors, and health self-efficacy should be investigated as an antecedent to health behaviors in our model, rather than a parallel mechanism. There is some precedent for this assertion, which could explain the lack of parallel and serial indirect effects for health behaviors and substantiation of serial indirect effects for health self-efficacy in model 3. Rosenstock et al. (1988) revised the health belief model to incorporate self-efficacy, along with perceived benefits and barriers, perceived vulnerability to a severe health threat, and sufficient motivation or health concern, as important explanatory factors in predicting health-related behavior among individuals with chronic illnesses (Rosenstock, 1966). As an example, in a sample of Ukrainian primary care patients with both chronic kidney disease and hypertension, self-efficacy accounted for 76% of the variance in quality of life associated with health behaviors (i.e., physical activity, diet, stress management, smoking, medication adherence, blood pressure control; Korzh et al., 2022). Further, in German primary care patients with heart failure, low self-efficacy predicted both poor lifestyle and medication adherence (Eisele et al., 2020).

Given that affective traits are known to influence health behaviors, the self-regulation resource model posits that affective (i.e., high positive and low negative affect) and social-cognitive variables (i.e., self-efficacy) work in conjunction to bolster the self-regulation capacity needed to perform health-promoting behaviors (Sirois, 2015). Gratitude is one possible positive affective trait that can be drawn upon, alongside health self-efficacy, to facilitate behavior change (Klibert et al., 2019; Wood et al., 2010). Self-efficacy mediated the effect of gratitude on medication adherence in heart failure patients (Cousin et al., 2020), and, in a systematic review, gratitude for the medical team and patient's donor, and self-efficacy, emerged as the primary factors contributing to medical self-management among kidney transplant recipients (Jamieson et al., 2016).

Multiple patterns emerged across models that warrant further discussion. In model 2, health behaviors were an independent mediator at all severity levels of sleep disturbances whereas, in model 3, health behaviors were not a significant mediator in serial pathways but emerged as an independent mediator of the relation between gratitude and sleep disturbances. This pattern of findings indicates that there is a strong independent influence of health behaviors on sleep. Engagement in both positive and negative health behaviors can have direct effects on sleep. For example, caffeine use can increase arousal and disrupt sleep onset, duration, and efficiency, and quality of slow-wave sleep (Clark and Landolt, 2017). Similarly, cocaine and nicotine intoxication can impair REM sleep, sleep onset time, and sleep duration (Garcia and Salloum, 2015). On the other hand, physical exercise and weight control can directly improve sleep quality, efficiency, duration, and onset latency (Alfaris et al., 2015; Rogers et al., 2017). Other health behaviors, such as a healthy diet, can help to regulate circadian rhythms through nutrients that affect the release of hormones, such as melatonin, growth hormone, and serotonin (Vernia et al., 2021).

The final notable finding, across models, was that, although gratitude and the first-order mediators (i.e., *M*_1_; health self-efficacy and/or health behaviors) predicted greater reductions in stress than anxiety and depression, the effect of stress on sleep disturbances was weaker than the effects of anxiety and depression. As well, serial mediation for the stress pathway occurred only in model 1, which was focused on health self-efficacy. Thus, although the broaden-and-build theory (Fredrickson, 2004) indicates that gratitude, health self-efficacy, and health behavior engagement may lead to stress reduction, these salubrious effects are not transmitted to sleep, as with anxiety and depression, unless health behaviors are excluded from the model and only health self-efficacy is considered. Because biological stress is considered a significant contributor to the development of anxiety and depression (Ross et al., 2017), physiological stress and, as a result, perceived stress, may exacerbate anxiety and depression, rather than escalating concurrently.

Overall, our findings indicate that gratitude has a greater effect on psychological distress through health self-efficacy than through health behaviors, and that health self-efficacy is the primary mechanism through which gratitude predicts reductions in psychological distress and downstream sleep problems.

### 4.1 Limitations and directions for future research

It is important that our findings are interpreted within the context of several study limitations. To begin, this study's cross-sectional design precludes determinations of causality (O'Laughlin et al., 2018) and, thus, bidirectionality of variables is a possibility. First, sleep disturbances and depression are known to have a bidirectional association in primary care patients (Bouwmans et al., 2017). This may occur because, in the context of a maladaptive stress response, a persistent feedback loop of anxiety and depressive symptoms, and sleep problems, can manifest (Gold, 2015). Additionally, sleep is considered a health behavior that, independently and through its impact on other health behaviors, such as diet and exercise, contributes to the development and progression of depression (Dzierzewski et al., 2014; Lopresti et al., 2013).

In adults, anxiety and depressive disorders predict unfavorable health behaviors, such as smoking, low physical activity, and poor diet (Difrancesco et al., 2022; Gall et al., 2016; Walsh et al., 2023), and self-efficacy and smoking behavior may be bidirectional (Clyde et al., 2019). In sum, given the current study's cross-sectional, survey-based design, future randomized controlled trials in which study variables are manipulated and prospective, longitudinal studies may help to elucidate these linkages.

Further, the use of self-report measures may limit external validity, as responses are affected by participants' understanding of the items, level of personal insight, and potential social desirability and response biases (Demetriou et al., 2015). Future studies should employ objective or physiological assessments, such as medical record reviews, actigraphy, polysomnography, and ecological momentary assessment, to improve validity (Jackson et al., 2018; Prince et al., 2020; Scarlett et al., 2021). Relatedly, the outcome variable of sleep disturbances was measured using only one item, item 3 of the PHQ-9, which precludes estimates of internal consistency and may not adequately capture a construct as multifaceted as sleep disturbances (Allen et al., 2022). However, this item has been validated in primary care patients as a brief screening tool for sleep disturbances (MacGregor et al., 2012). In future studies, utilizing a multidimensional measure, such as the Pittsburgh Sleep Quality Index (Buysse et al., 1989), may enhance reliability and validity and allow for the use of SEM or ordinary least squares regression at each stage of the model, simplifying interpretation of the regression results and potentially reducing measurement error.

Notably, as most participants (58%) reported zero or one medical condition, the results may not fully capture the experiences of individuals with multimorbidity. Nearly 40% of German adults experience multimorbidity (i.e., have two or more chronic medical conditions simultaneously), including half of adults between 50 and 59 years old, and 47% of adults between 40 and 49 years of age with low education levels (Puth et al., 2017). More chronic conditions predict development of stress, anxiety, and depression in representative population-based samples (Hajek and König, 2020; Liu et al., 2021), and sleep disturbances in primary care patients (Ullmann et al., 2022). Number of chronic diseases was covaried in all analyses and emerged as a significant predictor on almost all pathways of every model.

Finally, race and ethnicity statistics are not collected in German studies due to legal prohibitions and political factors, thus precluding inferences of generalizability for these demographic factors. Yet, it is likely that disparities exist, based on immigration research findings indicating that first-generation migrants in Germany have significantly higher rates of depression and generalized anxiety (Beutel et al., 2016). Future research conducted with additional demographic groups, chronic disease populations (e.g., cardiovascular, rheumatological, neurodegenerative), and sleep disorder populations (e.g., insomnia, sleep apnea, narcolepsy) is needed to improve generalizability and substantiate our findings across diverse groups.

### 4.2 Implications

Despite limitations, this study's novel findings may help to inform the selection of targeted clinical interventions to improve sleep in primary care patients. To begin, self-management interventions are effective for improving gratitude, health behaviors, self-efficacy, psychological distress, and sleep, including in individuals living with cancer (Martin et al., 2020), sedentary European adults with insomnia (Hartescu et al., 2015), and primary care patients from 10 practices in Germany (Zimmermann et al., 2016) and enrolled in a global cardiovascular risk reduction program (Prince et al., 2017). Importantly, according to a systematic review, to promote health self-efficacy, successful self-management programs in primary care should be tailored to meet individual needs, and should include independent symptom monitoring with self-treatment, regular communication with one's provider, stress management and coping strategies for addressing psychological distress, and promotion of responsibility for health behavior choices (Dineen-Griffin et al., 2019).

Further, systematic reviews and meta-analyses indicate beneficial effects of gratitude interventions for self-efficacy (Grant and Gino, 2010), stress, anxiety, and depression (Carr et al., 2021; Cregg and Cheavens, 2021), and subjective sleep quality (Boggiss et al., 2020). In a systematic review of positive psychological interventions (PPIs) in which 16 of 27 studies included gratitude-specific exercises, PPIs were responsible for favorable effects on health behaviors (Feig et al., 2022). Additional evidence-based interventions that may improve self-efficacy, health behaviors, psychological distress, and sleep disturbance include cognitive behavioral therapy (e.g., changing maladaptive thoughts to alter emotional responses) and acceptance and commitment therapy (e.g., fostering present-moment awareness, acceptance, and values-driven behavior), which can be efficaciously implemented in primary care settings (Arnold et al., 2022; Benninghoven et al., 2022; Hopko et al., 2009; Jonkers et al., 2012; Siengsukon et al., 2020). Behavioral activation for depression (i.e., identifying and intentionally scheduling enjoyable activities), a component of cognitive behavioral therapy, is particularly well-suited to cultivating engagement in salubrious behaviors, such as reducing problem drinking, facilitating smoking cessation, and improving physical activity and depression (May et al., 2024). In sum, as psychological distress and sleep disturbances are most often identified in primary care populations, providers have an opportunity to facilitate the initial catalyst of advantageous downstream effects that begins with promotion of gratitude.

### 4.3 Conclusion

In a sample of German primary care patients, health self-efficacy as a first-order mediator, and stress, anxiety, and depression, as parallel second-order mediators, serially mediated the relation between gratitude and sleep disturbances. In addition, health behaviors, and anxiety and depressive symptoms, functioned as serial mediators of the gratitude-sleep linkage. Finally, we found that health self-efficacy and health behaviors were not parallel first-order mediators, and only health self-efficacy and anxiety, and health self-efficacy and depression, serially mediated the relation between gratitude and sleep disturbances. Future prospective research utilizing objective assessments and across diverse samples is needed to substantiate these findings. However, despite limitations, this study provides preliminary support for the role of gratitude as a catalyst for health self-efficacy and health behaviors and, in turn, for its beneficial influence on psychological distress and sleep disturbances.

## Data Availability

The raw data supporting the conclusions of this article will be made available by the authors, without undue reservation.
