# Supplementary material for: Gratitude and sleep disturbance in primary care patients: the mediating roles of health self-efficacy, health behaviors, and psychological distress
Source: Front Sleep. 2025 Apr 17;4:1459854. doi: 10.3389/frsle.2025.1459854 (PMC12713842; doi:10.3389/frsle.2025.1459854)
Supplement: Supplementary file 1 [file Data_Sheet_1.pdf]

Appendix A
Supplementary Materials – Structural Equation Modeling

1. Method

Although path analysis with bootstrapped mediation analysis was deemed more appropriate and computationally feasible for estimating indirect effects while accounting for the measurement properties of the outcome variable, as a secondary analysis, we conducted structural equation modeling (SEM) with the lavaan package in R (Rosseel, 2012) to evaluate overall model fit. The outcome variable was initially treated as ordinal, but the assumption of proportional odds was violated for one of the predictors in each model (i.e., health self-efficacy in models 1 and 3 and health behaviors in model 2). To address this violation, we dichotomized the outcome, PHQ-9 item 3, using a cut score of 1 (i.e., sleep problems for several days over the past two weeks), which has been shown to provide the best balance of sensitivity and specificity in primary care patients (MacGregor et al., 2012). This allowed us to proceed with the analysis using logistic regression and SEM with an appropriate estimator for a categorical outcome, diagonally weighted least squares (DWLS). This dichotomization, while not ideal because it reduces statistical power and leads to less precise estimates of relationships, provided a way to handle the assumption violation without compromising the overall model structure.

Additionally, psychological distress was treated as a latent variable in the SEM model, comprised of our three second-order mediators, stress, anxiety, and depression, to account for measurement error and reduce model complexity. The latent variable was modeled using the total score of the stress, anxiety, and depression measures. We removed non-significant covariates (age, sex, education level, and number of chronic diseases) in a backward stepwise fashion, model-wide, unless model fit was improved. We used bias-corrected bootstrapping (10,000 samples) to estimate the 95% confidence intervals of the coefficients and mediation effects. The FDR method was used to adjust the *p*-values of coefficients for multiple comparisons with the p.adjust function (Benjamini and Hochberg, 1995; R Core Team, 2022).

2. Results

2.1 Model Fit

Model 1 demonstrated good fit to the data, $\chi^2_{(20)} = 34.402$, $p=.024$, Comparative Fit Index (CFI) = 0.987, Tucker-Lewis Index (TLI) = 0.994, Root Mean Square Error of Approximation (RMSEA) = 0.030 (90% CI [0.011, 0.047]), and Standardized Root Mean Square Residual (SRMR) = 0.025, based on established guidelines (Hu and Bentler, 1999).

Model 2 demonstrated good fit to the data, $\chi^2_{(18)} = 34.569$, $p=.011$, CFI = 0.984, TLI = 0.991, RMSEA = 0.034 (90% CI [0.016, 0.051]), SRMR = 0.024.

Model 3 demonstrated acceptable fit to the data, $\chi^2_{(22)} = 150.042$, $p=.000$, CFI = 0.908, TLI = 0.937, RMSEA = 0.086 (90% CI [0.073, 0.100]), SRMR = 0.086. After estimating the initial model, we evaluated modification indices to identify areas where the model could be improved.

We added covariances between the residuals of health self-efficacy and health behaviors, as these were consistent with the theoretical model and had the highest modification index (110.588). This modification improved model fit, as indicated by a reduction in the chi-square value and improvements in fit indices: $\chi^2_{(21)} = 38.883$, p=.010, CFI = 0.987, TLI = 0.991, RMSEA = 0.033 (90% CI [0.016, 0.049]), and SRMR = 0.027. No additional modifications were made after achieving acceptable fit indices, in line with recommendations to avoid overfitting (MacCallum, 1986).

2.2 Measurement Model

Factor loadings for all observed indicators were strong and significant at p<.001, ranging from 0.784 to 0.802 for stress, 0.788 to 0.799 for anxiety, and 0.832 to 0.841 for depression, indicating that psychological distress was adequately represented by these variables.

2.3 Structural Model

The SEM results reflected a similar pattern of findings as the path analyses. For model 1 (see Table S1), health self-efficacy as a first-order mediator, and psychological distress, as a second-order mediator, serially mediated the relation between gratitude and sleep disturbances ($a_1\ d_{21}\ b_2$ = -0.042, 95% CI [-0.064, -0.027], *SE* = 0.010). Additionally, specific indirect effects linking gratitude and sleep disturbances were observed via both health self-efficacy ($a_1\ b_1$ = -0.053, 95% CI [-0.087, -0.026], *SE* = 0.016) and psychological distress ($a_2\ b_2$ = -0.072, 95% CI [-0.125, -0.030], *SE* = 0.024). Overall, the total indirect effect (β = -0.168, 95% CI [-0.229, -0.112], *SE* = 0.030) and the total effect of gratitude on sleep disturbances (c = -0.103, 95% CI [-0.204, -0.005], *SE* = 0.050) were significant, while the direct effect was non-significant (c' = 0.064, 95% CI [-0.028, 0.157], *SE* = 0.047), indicating mediation.

Regarding model 2 (see Table S2), health behaviors as a first-order mediator, and psychological distress, as a second-order mediator, serially mediated the relation between gratitude and sleep disturbances ($a_1\ d_{21}\ b_2$ = -0.014, 95% CI [-0.026, -0.007], *SE* = 0.005). Additionally, specific indirect effects linking gratitude and sleep disturbances were observed via both health behaviors ($a_1\ b_1$ = -0.044, 95% CI [-0.074, -0.022], *SE* = 0.013) and psychological distress ($a_2\ b_2$ = -0.101, 95% CI [-0.154, -0.056], *SE* = 0.026). Overall, the total indirect effect (β = -0.159, 95% CI [-0.221, -0.103], *SE* = 0.031) and the total effect of gratitude on sleep disturbances (c = -0.107, 95% CI [-0.206, -0.008], *SE* = 0.050) were significant, while the direct effect was non-significant (c' = 0.053, 95% CI [-0.039, 0.142], *SE* = 0.046), indicating mediation.

Regarding model 3 (see Table S3), health self-efficacy ($a_1\ d_{21}\ b_3$ = -0.037, 95% CI [-0.058, -0.023], *SE* = 0.009) and health behaviors ($a_2\ d_{31}\ b_3$ = -0.013, 95% CI [-0.025, -0.006], *SE* = 0.005) as first-order mediators, and psychological distress, as a second-order mediator, serially mediated the relation between gratitude and sleep disturbances. Additionally, specific indirect effects linking gratitude and sleep disturbances were observed through health self-efficacy ($a_1\ b_1$ = -0.056, 95% CI [-0.092, -0.029], *SE* = 0.017), health behaviors ($a_2\ b_2$ = -0.044, 95% CI [-0.075, -0.022], *SE* = 0.014), and psychological distress ($a_3\ b_3$ = -0.050, 95% CI [-0.097, -0.012], *SE* = 0.022). Overall, the total indirect effect (β = -0.201, 95% CI [-0.269, -0.138], *SE* = 0.034) and the total effect of gratitude on sleep disturbances (c = -0.102, 95% CI [-0.200, -0.002], *SE* = 0.050)

were significant, while the direct effect was weaker but also significant (c' = 0.099, 95% CI [0.007, 0.191], *SE* = 0.048), indicating partial mediation.

After modifying model 3 (see Table S4) to add covariances between the residuals of health self-efficacy and health behaviors, improving model fit, health self-efficacy ($a_1\, d_{21}\, b_3$ = -0.036, 95% CI [-0.057, -0.022], *SE* = 0.009) and health behaviors ($a_2\, d_{31}\, b_3$ = -0.007, 95% CI [-0.017, -0.001], *SE* = 0.004) as first-order mediators, and psychological distress, as a second-order mediator, still serially mediated the relation between gratitude and sleep disturbances. However, specific indirect effects linking gratitude and sleep disturbances were observed through health behaviors ($a_2\, b_2$ = -0.037, 95% CI [-0.066, -0.017], *SE* = 0.013) and psychological distress ($a_3\, b_3$ = -0.070, 95% CI [-0.120, -0.028], *SE* = 0.024), but not health self-efficacy. The total indirect effect (β = -0.174, 95% CI [-0.238, -0.116], *SE* = 0.032) and the total effect of gratitude on sleep disturbances (c = -0.102, 95% CI [-0.200, -0.002], *SE* = 0.050) were significant, while the direct effect was non-significant (c' = 0.072, 95% CI [-0.017, 0.163], *SE* = 0.047), indicating mediation.

3. Interpretation

The SEM analysis provided support for the hypothesized models, demonstrating a similar pattern of effects and good model fit, despite dichotomizing the 4-level outcome variable. Adding covariances between the residuals of health self-efficacy and health behaviors to the third model improved model fit, providing support for their roles as first-order parallel mediators. Health self-efficacy no longer independently mediated the effect of gratitude on sleep disturbances once this covariance was accounted for, while the specific indirect effect of health behaviors remained significant, which is also consistent with our path analysis results.

4. References*

Hu, L., and Bentler, P. M. (1999). Cutoff criteria for fit indexes in covariance structure analysis: Conventional criteria versus new alternatives. *Structural Equation Modeling: A Multidisciplinary Journal.* 6, 1–55. doi: 10.1080/10705519909540118

MacCallum, R. (1986). Specification searches in covariance structure modeling. *Psychological Bulletin.* 100, 107–120. doi: 10.1037/0033-2909.100.1.107

**References listed are only those not included in main article.*

5 Tables

5.1 Table S1. Structural equation model for multiple mediation Model 1. The latent variable psychological distress (PD) is comprised of the total scores of the Perceived Stress Scale - 4, Generalized Anxiety Disorder 2-item, and Patient Health Questionnaire - 2. Depression (Patient Health Questionnaire - 2) is the reference variable. HSE = health self-efficacy. Estimates for pathways and mediation effects are standardized. CI = confidence interval, estimated by bias-corrected bootstrapping; *LL* = lower limit; *UL* = upper limit. *z* = *z*-score. *Adj. p* = Adjusted *p*-value (FDR correction).
[a] Completely standardized solution, with factor loadings calculated based on the variances of all the latent and observed variables in the model.

Pathway		Label	β	SE_β	95% CI β		*z*	*p*	*Adj. p*	Std. factor loading [a]
					LL	*UL*				
**Latent Variable**	**Observed Variable**									
PD	Depression		1.000	0.000	1.000	1.000				0.836
	Anxiety		1.021	0.048	0.936	1.117	21.257	0.000	0.000	0.798
	Stress		2.191	0.118	1.975	2.422	18.639	0.000	0.000	0.795
**Regressions**										
Sleep	PD	b2	0.446	0.062	0.338	0.548	7.203	0.000	0.000	0.475
	HSE	b1	-0.199	0.053	-0.301	-0.096	-3.745	0.000	0.000	-0.188
	Gratitude	c	0.064	0.047	-0.028	0.157	1.369	0.171	0.171	0.060
PD	HSE	d21	-0.358	0.044	-0.444	-0.271	-8.154	0.000	0.000	-0.318

Pathway		Label	β	SE_{β}	95% CI β		z	p	*Adj. p*	Std. factor loading [a]
					LL	*UL*				
	Gratitude	a2	-0.162	0.050	-0.266	-0.069	-3.233	0.001	0.002	-0.143
	age		-0.016	0.003	-0.023	-0.009	-4.685	0.000	0.000	-0.173
	gender		0.264	0.081	0.111	0.424	3.273	0.001	0.002	0.116
	conditions		0.241	0.032	0.180	0.305	7.523	0.000	0.000	0.321
HSE	Gratitude	a1	0.266	0.039	0.189	0.342	6.739	0.000	0.000	0.264
	age		0.008	0.003	0.002	0.014	2.625	0.009	0.011	0.096
	education		0.076	0.029	0.018	0.132	2.600	0.009	0.011	0.105
	conditions		-0.200	0.024	-0.249	-0.154	-8.220	0.000	0.000	-0.300
**Thresholds**										
Sleep	: 1		0.946	0.348	0.266	1.633	2.723	0.006	0.008	0.908
**Variances**										
Depression			0.532	0.057	0.430	0.649	9.294	0.000	0.000	0.302
Anxiety			0.734	0.066	0.623	0.878	11.132	0.000	0.000	0.364
Stress			3.431	0.322	2.843	4.086	10.646	0.000	0.000	0.367
Sleep			0.735	0.000	0.735	0.735				0.677

Pathway	Label	β	SE_{β}	95% CI β		*z*	*p*	*Adj. p*	Std. factor loading [a]
				LL	*UL*				
HSE		0.778	0.039	0.709	0.864	19.956	0.000	0.000	0.799
PD		0.829	0.081	0.690	1.012	10.250	0.000	0.000	0.673
**Mediation effects**									
Gratitude → HSE → PD → Sleep	a1*d21*b2	-0.042	0.010	-0.064	-0.027	-4.335	0.000	0.000	-0.040
Gratitude → HSE → Sleep	a1*b1	-0.053	0.016	-0.087	-0.026	-3.340	0.001	0.001	-0.050
Gratitude → PD → Sleep	a2*b2	-0.072	0.024	-0.125	-0.030	-2.986	0.003	0.004	-0.068
Total	*c*	-0.103	0.050	-0.204	-0.005	-2.058	0.040	0.044	-0.097
Total indirect		-0.168	0.030	-0.229	-0.112	-5.530	0.000	0.000	-0.158
Direct	c'	0.064	0.047	-0.028	0.157	1.370	0.171	0.171	0.060
		R^2							
Depression		0.698							
Anxiety		0.636							
Stress		0.633							

Pathway	Label	β	$SE_{β}$	95% CI β		z	p	*Adj. p*	Std. factor loading [a]
				LL	*UL*				
Sleep		0.323							
HSE		0.201							
PD		0.327							

5.2 Table S2. Structural equation model for multiple mediation Model 2. The latent variable psychological distress (PD) is comprised of the total scores of the Perceived Stress Scale - 4, Generalized Anxiety Disorder 2-item, and Patient Health Questionnaire - 2. Depression (Patient Health Questionnaire - 2) is the reference variable. HB = health behaviors. Estimates for pathways and mediation effects are standardized. CI = confidence interval, estimated by bias-corrected bootstrapping; *LL* = lower limit; *UL* = upper limit. z = z-score. *Adj. p* = Adjusted p-value (FDR correction).
[a] Completely standardized solution, with factor loadings calculated based on the variances of all the latent and observed variables in the model.

Pathway		Label	β	$SE_{β}$	95% CI β		z	p	*Adj. p*	Std. factor loading [a]
					LL	*UL*				
**Latent Variable**	**Observed Variable**									
PD	Depression		1.000	0.000	1.000	1.000				0.841
	Anxiety		1.014	0.048	0.925	1.107	21.144	0.000	0.000	0.799

Pathway		Label	β	$SE_β$	95% CI β		*z*	*p*	*Adj. p*	Std. factor loading [a]
					LL	*UL*				
	Stress		2.150	0.119	1.930	2.385	17.988	0.000	0.000	0.784
**Regressions**										
Sleep	PD	b2	0.433	0.057	0.328	0.529	7.590	0.000	0.000	0.454
	HB	b1	-0.295	0.047	-0.382	-0.203	-6.307	0.000	0.000	-0.276
	Gratitude	c	0.053	0.046	-0.039	0.142	1.146	0.252	0.252	0.049
	age		0.010	0.004	0.001	0.019	2.343	0.019	0.023	0.117
	conditions		0.065	0.037	-0.006	0.137	1.775	0.076	0.084	0.091
PD	HB	d21	-0.223	0.042	-0.307	-0.143	-5.315	0.000	0.000	-0.199
	Gratitude	a2	-0.233	0.053	-0.341	-0.134	-4.414	0.000	0.000	-0.205
	age		-0.013	0.004	-0.021	-0.006	-3.385	0.001	0.001	-0.140
	gender		0.287	0.081	0.131	0.446	3.564	0.000	0.001	0.126
	conditions		0.279	0.034	0.215	0.345	8.307	0.000	0.000	0.372
HB	Gratitude	a1	0.149	0.037	0.077	0.223	3.992	0.000	0.000	0.147
	age		0.027	0.003	0.021	0.033	8.977	0.000	0.000	0.327
	education		0.077	0.028	0.024	0.133	2.794	0.005	0.007	0.106
	conditions		-0.096	0.026	-0.145	-0.045	-3.750	0.000	0.000	-0.144

Pathway	Label	β	SE_β	95% CI β		*z*	*p*	*Adj. p*	Std. factor loading [a]
				LL	*UL*				
**Thresholds**									
Sleep : 1		1.423	0.358	0.732	2.134	3.972	0.000	0.000	1.340
**Variances**									
Depression		0.512	0.059	0.408	0.639	8.712	0.000	0.000	0.292
Anxiety		0.720	0.066	0.609	0.862	10.968	0.000	0.000	0.361
Stress		3.595	0.334	2.979	4.269	10.771	0.000	0.000	0.386
Sleep		0.698	0.000	0.698	0.698				0.619
HB		0.867	0.040	0.796	0.957	21.577	0.000	0.000	0.878
PD		0.903	0.086	0.752	1.094	10.444	0.000	0.000	0.728
**Mediation effects**									
Gratitude → HB → PD → Sleep	a1*d21*b2	-0.014	0.005	-0.026	-0.007	-2.873	0.004	0.005	-0.013
Gratitude → HB → Sleep	a1*b1	-0.044	0.013	-0.074	-0.022	-3.351	0.001	0.001	-0.041
Gratitude → PD → Sleep	a2*b2	-0.101	0.026	-0.154	-0.056	-3.947	0.000	0.000	-0.093

Pathway	Label	β	$SE_β$	95% CI β		*z*	*p*	*Adj. p*	Std. factor loading [a]
				LL	*UL*				
Total	*c*	-0.107	0.050	-0.206	-0.008	-2.114	0.034	0.039	-0.098
Total indirect		-0.159	0.031	-0.221	-0.103	-5.170	0.000	0.000	-0.147
Direct	c'	0.053	0.046	-0.039	0.142	1.147	0.251	0.252	0.049
		R^2							
Depression		0.708							
Anxiety		0.639							
Stress		0.614							
Sleep		0.381							
HB		0.122							
PD		0.272							

5.3 **Table S3.** Structural equation model for multiple mediation Model 3. The latent variable psychological distress (PD) is comprised of the total scores of the Perceived Stress Scale - 4, Generalized Anxiety Disorder 2-item, and Patient Health Questionnaire - 2. Depression (Patient Health Questionnaire - 2) is the reference variable. HB = health behaviors. Estimates for pathways and mediation effects are standardized. CI = confidence interval, estimated by bias-corrected bootstrapping; *LL* = lower limit; *UL* = upper limit. *z* = *z*-score. *Adj. p* = Adjusted *p*-value (FDR correction).

[a] Completely standardized solution, with factor loadings calculated based on the variances of all the latent and observed variables in the model.

Pathway		Label	β	$SE_β$	95% CI β		*z*	*p*	*Adj. p*	Std. factor loading [a]
					LL	*UL*				
**Latent Variable**	**Observed Variable**									
PD	Depression		1.000	0.000	1.000	1.000				0.833
	Anxiety		1.014	0.053	0.927	1.109	19.073	0.000	0.000	0.788
	Stress		2.233	0.123	2.012	2.475	18.173	0.000	0.000	0.802
**Regressions**										
Sleep	HSE	b1	-0.214	0.055	-0.318	-0.109	-3.917	0.000	0.000	-0.202
	HB	b2	-0.300	0.050	-0.389	-0.200	-5.948	0.000	0.000	-0.284
	PD	b3	0.387	0.073	0.266	0.499	5.272	0.000	0.000	0.405
	Gratitude	c	0.099	0.048	0.007	0.191	2.075	0.038	0.041	0.092
	age		0.011	0.004	0.003	0.020	2.549	0.011	0.014	0.130
PD	HSE	d21	-0.364	0.044	-0.452	-0.278	-8.300	0.000	0.000	-0.326
	HB	d31	-0.232	0.042	-0.315	-0.152	-5.593	0.000	0.000	-0.210
	Gratitude	a3	-0.129	0.051	-0.232	-0.031	-2.512	0.012	0.015	-0.115
	age		-0.010	0.004	-0.017	-0.003	-2.625	0.009	0.011	-0.108
	gender		0.269	0.081	0.112	0.428	3.325	0.001	0.001	0.119

Pathway		Label	β	SE_{β}	95% CI β		*z*	*p*	*Adj. p*	Std. factor loading [a]
					LL	*UL*				
	education		0.042	0.031	-0.017	0.103	1.371	0.170	0.175	0.052
	conditions		0.215	0.033	0.150	0.280	6.513	0.000	0.000	0.288
HSE	Gratitude	a1	0.263	0.040	0.186	0.340	6.608	0.000	0.000	0.262
	age		0.008	0.003	0.002	0.014	2.773	0.006	0.008	0.099
	education		0.084	0.024	0.036	0.131	3.477	0.001	0.001	0.117
	conditions		-0.199	0.024	-0.248	-0.153	-8.180	0.000	0.000	-0.298
HB	Gratitude	a2	0.148	0.038	0.076	0.224	3.901	0.000	0.000	0.146
	age		0.027	0.003	0.021	0.033	8.703	0.000	0.000	0.323
	education		0.079	0.025	0.031	0.129	3.154	0.002	0.002	0.110
	conditions		-0.094	0.025	-0.143	-0.044	-3.708	0.000	0.000	-0.140
**Thresholds**										
Sleep	: 1		1.585	0.358	0.903	2.285	4.432	0.000	0.000	1.511
**Variances**										
Depression			0.533	0.059	0.430	0.658	9.027	0.000	0.000	0.306
Anxiety			0.757	0.073	0.639	0.907	10.436	0.000	0.000	0.378
Stress			3.336	0.336	2.721	4.019	9.920	0.000	0.000	0.357

Pathway	Label	β	SE_{β}	95% CI β		*z*	*p*	*Adj. p*	Std. factor loading [a]
				LL	*UL*				
Sleep		0.656	0.000	0.656	0.656				0.597
HSE		0.775	0.039	0.707	0.860	19.936	0.000	0.000	0.797
HB		0.866	0.040	0.797	0.955	21.450	0.000	0.000	0.879
PD		0.765	0.082	0.625	0.948	9.332	0.000	0.000	0.634
**Mediation effects**									
Gratitude → HSE → PD → Sleep	a1*d21*b3	-0.037	0.009	-0.058	-0.023	-3.916	0.000	0.000	-0.035
Gratitude → HB → PD → Sleep	a2*d31*b3	-0.013	0.005	-0.025	-0.006	-2.711	0.007	0.009	-0.012
Gratitude → HSE → Sleep	a1*b1	-0.056	0.017	-0.092	-0.029	-3.410	0.001	0.001	-0.053
Gratitude → HB → Sleep	a2*b2	-0.044	0.014	-0.075	-0.022	-3.247	0.001	0.002	-0.042
Gratitude → PD → Sleep	a3*b3	-0.050	0.022	-0.097	-0.012	-2.276	0.023	0.027	-0.047
Total	*c*	-0.102	0.050	-0.200	-0.002	-2.022	0.043	0.045	-0.096
Total indirect		-0.201	0.034	-0.269	-0.138	-5.835	0.000	0.000	-0.188

Pathway	Label	β	SE_{β}	95% CI β		*z*	*p*	*Adj. p*	Std. factor loading [a]
				LL	*UL*				
Direct	c'	0.099	0.048	0.007	0.191	2.077	0.038	0.041	0.092
		R²							
Depression		0.694							
Anxiety		0.622							
Stress		0.643							
Sleep		0.403							
HSE		0.203							
HB		0.121							
PD		0.366							

5.4 Table S4. Structural equation model for multiple mediation Model 3, after model improvement. The latent variable psychological distress (PD) is comprised of the total scores of the Perceived Stress Scale - 4, Generalized Anxiety Disorder 2-item, and Patient Health Questionnaire - 2. Depression (Patient Health Questionnaire - 2) is the reference variable. HB = health behaviors. Estimates for pathways and mediation effects are standardized. CI = confidence interval, estimated by bias-corrected bootstrapping; *LL* = lower limit; *UL* = upper limit. *z* = *z*-score. *Adj. p* = Adjusted *p*-value (FDR correction). Bolded *p*-values indicate those that became non-significant after adjustment.

[a] Completely standardized solution, with factor loadings calculated based on the variances of all the latent and observed variables in the model.

Pathway		Label	β	SE_{β}	95% CI β		*z*	*p*	*Adj. p*	Std. factor loading [a]
					LL	*UL*				
**Latent Variable**	**Observed Variable**									
PD	Depression		1.000	0.000	1.000	1.000				0.832
	Anxiety		1.014	0.053	0.927	1.109	19.103	0.000	0.000	0.788
	Stress		2.234	0.123	2.014	2.477	18.208	0.000	0.000	0.802
**Regressions**										
Sleep	HSE	b1	-0.091	0.060	-0.202	0.018	-1.523	0.128	0.137	-0.086
	HB	b2	-0.250	0.057	-0.351	-0.145	-4.400	0.000	0.000	-0.239
	PD	b3	0.439	0.069	0.329	0.544	6.348	0.000	0.000	0.463
	Gratitude	c	0.072	0.047	-0.017	0.163	1.549	0.121	0.134	0.068
	age		0.010	0.005	0.001	0.019	2.203	0.028	0.035	0.115
PD	HSE	d21	-0.316	0.049	-0.415	-0.223	-6.440	0.000	0.000	-0.283
	HB	d31	-0.108	0.046	-0.196	-0.017	-2.364	0.018	0.024	-0.098
	Gratitude	a3	-0.160	0.050	-0.260	-0.065	-3.191	0.001	0.002	-0.143
	age		-0.014	0.004	-0.021	-0.007	-3.725	0.000	0.000	-0.148
	gender		0.269	0.081	0.112	0.427	3.325	0.001	0.001	0.119

Pathway		Label	β	$SE_β$	95% CI β		*z*	*p*	*Adj. p*	Std. factor loading [a]
					LL	*UL*				
	education		0.027	0.030	-0.031	0.086	0.889	0.374	0.374	0.033
	conditions		0.238	0.032	0.176	0.300	7.478	0.000	0.000	0.319
HSE	Gratitude	a1	0.263	0.040	0.186	0.340	6.608	0.000	0.000	0.262
	age		0.008	0.003	0.002	0.014	2.773	0.006	0.007	0.099
	education		0.084	0.024	0.036	0.132	3.453	0.001	0.001	0.117
	conditions		-0.198	0.025	-0.247	-0.152	-8.058	0.000	0.000	-0.297
HB	Gratitude	a2	0.148	0.038	0.076	0.224	3.901	0.000	0.000	0.146
	age		0.027	0.003	0.021	0.033	8.703	0.000	0.000	0.323
	education		0.081	0.025	0.032	0.131	3.182	0.001	0.002	0.111
	conditions		-0.094	0.026	-0.145	-0.044	-3.695	0.000	0.000	-0.140
**Covariances**										
HSE	HB		0.341	0.032	0.282	0.406	10.729	0.000	0.000	0.416
**Thresholds**										
Sleep	: 1		1.367	0.362	0.669	2.063	3.777	0.000	0.000	1.311
**Variances**										
Depression			0.536	0.059	0.432	0.660	9.080	0.000	0.000	0.307

Pathway	Label	β	$SE_{β}$	95% CI β		*z*	*p*	*Adj. p*	Std. factor loading [a]
				LL	*UL*				
Anxiety		0.759	0.073	0.642	0.910	10.467	0.000	0.000	0.379
Stress		3.341	0.336	2.725	4.022	9.944	0.000	0.000	0.357
Sleep		0.681	0.000	0.681	0.681				0.627
HSE		0.775	0.039	0.707	0.861	19.881	0.000	0.000	0.798
HB		0.867	0.041	0.797	0.956	21.408	0.000	0.000	0.879
PD		0.802	0.081	0.663	0.981	9.921	0.000	0.000	0.664
**Mediation effects**									
Gratitude → HSE → PD → Sleep	a1*d21*b3	-0.036	0.009	-0.057	-0.022	-3.917	0.000	0.000	-0.034
Gratitude → HB → PD → Sleep	a2*d31*b3	-0.007	0.004	-0.017	-0.001	-1.815	0.070	0.081	-0.007
Gratitude → HSE → Sleep	a1*b1	-0.024	0.016	-0.056	0.004	-1.470	0.142	0.149	-0.023
Gratitude → HB → Sleep	a2*b2	-0.037	0.013	-0.066	-0.017	-2.857	0.004	0.006	-0.035

Pathway	Label	β	SE_{β}	95% CI β		*z*	*p*	*Adj. p*	Std. factor loading [a]
				LL	*UL*				
Gratitude → PD → Sleep	a3*b3	-0.070	0.024	-0.120	-0.028	-2.930	0.003	0.005	-0.066
Total	*c*	-0.102	0.050	-0.200	-0.002	-2.022	0.043	**0.052**	-0.096
Total indirect		-0.174	0.032	-0.238	-0.116	-5.452	0.000	0.000	-0.164
Direct	c'	0.072	0.047	-0.017	0.163	1.550	0.121	0.134	0.068
		R^2							
Depression		0.693							
Anxiety		0.621							
Stress		0.643							
Sleep		0.373							
HSE		0.202							
HB		0.121							
PD		0.336							
